# Differential microRNA expression in human placentas of term intra-uterine growth restriction that regulates target genes mediating angiogenesis and amino acid transport

**DOI:** 10.1371/journal.pone.0176493

**Published:** 2017-05-02

**Authors:** Shanthie Thamotharan, Alison Chu, Katie Kempf, Carla Janzen, Tristan Grogan, David A. Elashoff, Sherin U. Devaskar

**Affiliations:** 1 Department of Pediatrics, Division of Neonatology & Developmental Biology, David Geffen School of Medicine at UCLA, Los Angeles, California, United States of America; 2 Department of Obstetrics and Gynecology, David Geffen School of Medicine at UCLA, Los Angeles, California, United States of America; 3 Department of Medicine Statistics Core, David Geffen School of Medicine, University of California, Los Angeles, California, United States of America; Hopital Robert Debre, FRANCE

## Abstract

Placental insufficiency leading to intrauterine growth restriction (IUGR) demonstrates perturbed gene expression affecting placental angiogenesis and nutrient transfer from mother to fetus. To understand the post-transcriptional mechanisms underlying such placental gene expression changes, our objective was to identify key non-coding *microRNAs* that express biological function. To this end, we initially undertook microarrays targeting microRNAs in a small sub-set of placentas of appropriate (AGA) versus small for gestational age (SGA) weight infants, and observed up-regulation of 97 miRs and down-regulation of 44 miRs in SGA versus AGA. In a larger cohort of samples (AGA, n = 21; SGA, n = 11; IUGR subset, n = 5), we validated by qRT-PCR differential expression of three specific microRNAs (*miR-10b*, *-363 and -149*) that target genes mediating angiogenesis and nutrient transfer. Validation yielded an increase in *miR-10b* and *-363* expression of ~2.5-fold (p<0.02 each) in SGA versus AGA, and of ~3-fold (p<0.005) in IUGR versus AGA, with no significant change despite a trending increase in *miR-149*. To further establish a cause-and-effect paradigm, employing human HTR8 trophoblast cells, we assessed the effect of nutrient deprivation on miR expression and inhibition of endogenous miRs on target gene expression. *In-vitro* nutrient deprivation (~50%) increased the expression of *miR-10b* and *miR-149* by 1.5-fold (p<0.02) while decreasing *miR-363* (p<0.0001). Inhibition of endogenous miRs employing antisense sequences against *miR-10b*, *-363* and *-149* revealed an increase respectively in the expression of the target genes *KLF-4* (transcription factor which regulates angiogenesis), *SNAT1* and *2* (sodium coupled neutral amino acid transporters) and *LAT2* (leucine amino acid transporter), which translated into a similar change in the corresponding proteins. Finally to establish functional significance we performed dual-luciferase reporter assays with 3’-insertion of *miR-10b* alone and observed a ~10% reduction in the 5’-luciferase activity versus the control. Lastly, we further validated by microarray and employing MirWalk software that the pathways and target genes identified by differentially expressed miRs in SGA/IUGR compared to AGA are consistent in a larger cohort. We have established the biological significance of various miRs that target common transcripts mediating pathways of importance, which are perturbed in the human IUGR placenta.

## Introduction

Intrauterine growth restriction (IUGR) is a common condition of pregnancy, affecting up to 5–10% of pregnancies worldwide with myriad causes, but remaining poorly understood. IUGR is defined by the American Congress of Obstetricians and Gynecologists as the inability of a fetus to meet its growth potential, and is widely characterized by a fetus with birthweight <10% for gestational age and often with evidence of third trimester growth deceleration or blood flow abnormalities as measured by prenatal ultrasound [1,2]. Small-for-gestational age (SGA), in distinction, is defined as a baby with a birthweight <10%, with or without evidence of third trimester ultrasound abnormalities. Thus SGA may encompass IUGR babies as well. However, in contrast to SGA, IUGR puts the newborn at increased risk for perinatal morbidity and mortality [3,4] and worse neurodevelopmental outcomes [5–7]. As adults, these offspring have increased risk of cardio-metabolic disease, including obesity, hypertension, and non-insulin dependent diabetes mellitus [8–10].

Most often, IUGR results from placental insufficiency, whereby a diminished supply of nutrients and oxygen to the growing fetus results in impaired somatic growth. Disruption to the normal structure and function of the placenta results in a significantly altered fetal environment, and therefore, the expression of key placental genes that determine placental size, vascularity, and function will regulate, in turn, fetal growth [11,12]. Gene expression is regulated at multiple levels. Our group and others have demonstrated transcriptional changes in IUGR mouse and human placentas [13,14], and more recently, a role for post-transcriptional changes has also emerged [15–18]. In particular, certain non-coding microRNAs were identified to be perturbed.

MicroRNAs are small, ~22 nucleotide, non-coding RNAs that affect post-transcriptional gene regulation by targeting a network of mRNAs in a variety of diverse biological processes [19]. Not only does the placenta express miRNAs, there also appears to be particular miRNA clusters distinct to the placenta, and moreover, miRNA “signatures” may be associated with certain placental pathologies, including placental insufficiency associated with intrauterine growth restriction or preeclampsia [20–24].

Given these previously reported findings, we undertook the present investigation to determine whether miRNA signatures are distinct in intrauterine growth restricted (IUGR) placentas compared to appropriate-for-gestational age (AGA) infants, and to assess potential biologic function of certain differentially expressed miRNAs *in vitro*. In our experiments, we found that whole placental microRNA expression does differ in IUGR compared to their AGA counterparts, initially in a sub-set of limited numbers using microarray analyses, followed by validation of specific miRs expression in a larger cohort. These specific miRs were chosen based on their biological significance in placental well being, with potential target genes that affect pathways of significance to vascularization, metabolism and cellular growth and maturation. Using an *in vitro* model of nutrient deficiency to mimic that seen in IUGR, we provide evidence that specifically selected miRNAs *(-10b*, *-363*, *-149)* are differentially expressed by trophoblasts. Furthermore, inhibition of these miRNAs results in increased expression of their target genes, which include those important for angiogenesis and nutrient transfer. We lastly further validated that our target genes of interest and their pathways affected by differential microRNA expression in IUGR placentas are consistent in a larger cohort. Overall, these selected miRNAs affect potential biologic pathways important for placental blood flow and nutrient provision to the fetus. These experimental results provide insights into post-transcriptional pathophysiologic mechanisms of placental disease in IUGR, and identify potential biomarkers that could lead to improved diagnostic and perhaps therapeutic options.

## Materials and methods

### 1. Study participants and placental specimen collection

Collection and processing of the placentas used for this study have been described previously [14] in detail. This study received Institutional Review Board Approval from the University of California-Los Angeles, and placentas and clinical information were collected only after obtaining maternal consents. Briefly, informed consent was obtained from women with term (>37 weeks gestational age) appropriate-for gestational age fetuses (n = 21), or with concern for SGA or late-onset IUGR (n = 10). Appropriate for gestational age (AGA) placentas were defined as the newborn infant demonstrating a birth weight between the 10^th^ and 90^th^ percentiles. Small for gestational age was defined as a newborn weight <10%ile for gestational age, without abnormal prenatal ultrasound findings of growth deceleration and/or abnormal Doppler blood flow measurements. IUGR was defined by newborn weight ≤10^th^ percentile for gestational age and a trajectory of fetal growth deceleration in utero, diagnosed by ultrasound, and abnormal Doppler blood flow measurements. The pregnancies complicated by SGA or fetal growth restriction were not associated with known maternal systemic disease, multiparity, pre-eclampsia, infection, or known fetal chromosomal, genetic, or congenital abnormalities. These conditions served as exclusion criteria in this study. Placentas were collected at the time of delivery, and immediately dissected as follows: the decidual layer, basal plate, and chorionic surface and membranes were removed by sharp dissection, and placental fragments were obtained at the middle of the initial placental depth. Placental samples were snap frozen in liquid nitrogen and stored at -80°C.

#### 1.1 miRNA isolation

Total cellular microRNA from human placentas was purified using the Qiagen miRNeasy Mini Kit (Qiagen, Valencia, CA) per manufacturer’s protocol. The tissue was prepared by adding 700 μl QIAzol Lysis Reagent to 50 mg of placental tissue and homogenized for one minute using the Omni tissue homogenizer (OMNI International, Kennesaw, GA). After elution of the microRNA, the Qiagen RNeasy MinElute Cleanup Kit (Qiagen) was used to remove any contaminating DNA and concentrate the samples. RNA concentration and quality was measured using the NanoDrop 2000c spectrophotometer (ThermoFisher Scientific, Waltham, MA, USA).

#### 1.2 MiRNA microarray

Three AGA and three IUGR placental samples were initially arbitrarily selected to retain variability within the samples collected and subjected to genome-wide microarray analysis, performed by the Clinical Microarray Core facility at UCLA, using the Affymetrix GeneChip miRNA 3.0 Array (Affymetrix, Santa Clara, CA). This array chip contains 25,015 probe sets covering 153 organisms. Of these probe sets 5,617 are designated as human “hsa” probes, while 1,789 are specific for mature human miRNAs. The data obtained was initially analyzed using the Express Console^™^ Software version 1.2 (Affymetrix). For this study, we focused primarily on mature human miRNAs, labeled “hsa”. Once differentially expressed genes were detected (as defined in the “Statistical Analysis” section), the data set was re-analyzed.

#### 1.3 Quantitative real time polymerase chain reaction (RT-qPCR)

Reverse transcription was carried out using the miRCURY LNA^™^ Universal RT microRNA PCR protocol (Exiqon, Woburn, MA), per manufacturer’s instructions. A spike-in control was added to each sample prior to reverse transcription. A total of 20 ng of miRNA was reverse transcribed for each individual sample (n = 7-11/group). The RT product was then diluted by a factor of 80 and 4 μl RT product was combined with 5 μl SYBR^®^ Green master mix and 1 μl PCR primer mix to a total volume of 10 μl. SNORD48 was used as the endogenous control and control primers were used to detect the added spike-in. Primer mixes for SNORD48 (hsa), hsamir-363-3p, hsa-mir-10b-5p, and hsa-mir-149-5p were all purchased from Exiqon (Woburn, MA). Each sample was run in triplicate using forward and reverse primers that detect the miRNA of interest, primers for the endogenous SNORD48, and primers for the control spike-in. Quantification was carried out using the ABI 7900HT system (Applied Biosystems, Foster City, CA). The fold increase relative to control samples was determined by the comparative Ct method of calculations described by Livak [25].

#### 1.4 Western immunoblotting

Briefly, tissue homogenates from whole placental samples were solubilized in 50 mM Tris, pH 6.8, containing 2% SDS. Protein concentration was determined by using the Bio-Rad dye-binding assay. Western blotting was performed as described previously (n = 5-10/group) [26]. Briefly, the solubilized protein homogenates (50 μg) were subjected to electrophoresis on 10% SDS-polyacrylamide gels and transferred to nitrocellulose membranes (Transblot; Bio-Rad, Hercules, CA). The following primary antibodies were used for signal detection: *LAT2* antibody (Santa Cruz, Dallas, TX; ~58 kDA; 1:500 dilution) and *KLF4* antibody (Cell Signaling, Danvers, MA; ~65 kDA; 1:500 dilution). Anti-vinculin antibody (Sigma-Aldrich, St. Louis, MO, 1:5000) was used to detect endogenous vinculin, which served as an internal control for inter-lane loading variability [26]. The quantification of protein bands was performed by densitometry using ImageQuant software (GE Healthcare). The optical density was corrected for inter-lane loading variability using an internal control. For all bands, resultant optical density was ensured as linear to the loading protein concentrations, and the intensity of the protein bands was assessed by densitometry using the Scion Image software program [27].

### 2. Trophoblast cell culture

Two separate cell lines were employed for these studies. The human extravillous trophoblast cell line HTR-8 (gifted by Ravi Jhaveri, Duke University) was cultured in RPMI 1640 media (Invitrogen^™^, Life Technologies, Grand Island, NY) containing 10% fetal bovine serum (Invitrogen^™^, Life Technologies), 100 U/ml penicillin and 100 μg/ml streptomycin, and grown (Thermo Fisher Scientific, Waltham, MA) in 5% CO_2_ and 95% air at 37°C, constituting control conditions. This cell line depicted the invasive properties of trophoblastic cells evident during the first trimester of pregnancy. The second cell line employed was the human choriocarcinoma BeWo cell line that depicts syncytial properties with barrier function characteristically seen during the second and third trimesters of pregnancy [28]. BeWo cells were cultured and maintained under similar conditions as the HTR8 cell line.

#### 2.1 miRNA isolation

miRNA was isolated as described in section 1.1, except that in the case of HTR8 and BeWo cells, 1.8 X 10^6^ cells/ml in 700 μl lysis buffer served as the starting point for extraction of miRNA following the same protocol. In addition, for these samples, on-column DNA digestion was undertaken.

#### 2.2 Nutrient restriction

To mimic nutrient restriction, HTR-8 cells were grown under 50% media-restricted conditions that was pre-determined to be optimal for these cells, where the RPMI 1640 media with all its additives was diluted with an equal volume of tissue culture grade PBS with 23.8 mM sodium bicarbonate, and the cells maintained in 5% CO_2_ and 95% air at 37°C. Control and media-restricted cellular miRNA was extracted at 0h (baseline), 1h, 3h, 6h and 24h of nutrient restriction.

#### 2.3 Inhibition of endogenous miRs

SiRNA-mediated knockdown experiments were performed as previously described [29]. Briefly, to attain RNAi, 5x10^6^ freshly seeded cells were transfected with targeting *miR-10b* inhibitor, *miR-363* inhibitor, *miR-149* inhibitor (Thermofisher Scientific, Carlsbad, CA) or the negative control at a final concentration of 30 nM, using HiPerfect Transfection Kit (Qiagen, Valencia, CA), according to the manufacturer’s instructions. Cells were harvested at previously determined optimal times of 48 hours (for RNA extraction) and 72 hours (for protein studies) after transfection. Endogenous expression of the miRNA of interest and its downstream targets were assessed using qRT-PCR or Western blotting. For all miRNAs of interest (*-10b*, *-363*, *-149*), successful inhibition was verified by quantification of miRNA gene expression at control conditions and after inhibition by qRT-PCR, and compared to the negative controls provided by the manufacturer (n = 3-6/condition for each miRNA comparison). Downstream targets for each miRNA of interest were identified using the TargetScan program. For these studies, we focused on target genes mediating angiogenesis, cell proliferation or nutrient transfer, as these processes are essential for placental function and potentially altered in intrauterine growth restriction.

**2.3.1 Evaluation of miRNA downstream targets by qRT-PCR and western blotting**: For *miRNA-10b*, gene expression of downstream targets, *E-cadherin* (n = 5-6/group), *HoxD* (n = 8/group) and *KLF-4* (n = 8/group), were assessed by qRT-PCR after inhibition of the endogenous miR. Protein expression was also assessed by western blotting for *E-cadherin* and *HoxD10* (n = 4-5/group) after miRNA-10b inhibition.

For *miRNA-363*, gene expression of downstream targets, *EGF*, *Glut-3*, *insulin receptor*, *SNAT1* and *SNAT2* (n = 3-6/group), was evaluated by qRT-PCR. SNAT1 and SNAT2 protein expression was assessed by western blotting (n = 3-6/group).

For *miRNA-149*, *LAT2* gene and protein expression was assessed by qRT-PCR and western blotting, respectively.

For quantitative real time polymerase chain reaction (RT-qPCR) studies, qRT-PCR was performed as described in section 1.3, with the following modifications for miRNA gene expression and downstream targets. Primers and probe sequences and annealing temperatures included in S1 Table, except for *KLF4*, which was detected using Assay ID Hs 00358836 (Life Technologies, Carlsbad, US). Each sample was run in triplicate using forward and reverse primers that detect the miRNA of interest, and primers for the control spike-in. Quantification was carried out using the ABI 7900HT system (Applied Biosystems, Foster City, CA). The fold increase relative to control samples was determined by the comparative Ct method of calculations described by Livak [25].

For western blot analysis, protein was isolated from HTR-8 cells and the BeWo cells by washing cells with PBS, treating them with trypsin, and pelleting by centrifugation. Cells were then lysed with lysis buffer. Protein concentration was determined by using the Bio-Rad dye-binding assay. Western blotting was performed as described previously (n = 3/group) [26]. Briefly, the solubilized protein homogenates (20 μg) were subjected to electrophoresis on 10% SDS-polyacrylamide gels and transferred to nitrocellulose membranes (Transblot; Bio-Rad, Hercules, CA). The following primary antibodies were used for signal detection: E-cadherin (cat# NBP1-19051, Novus Biologicals, Littleton, CO; ~135 kDA; 1:500 dilution), HOXD10 (cat# NBP1-28476, Novus Biologicals, Littleton, CO; ~64.5kD; 1:1000 dilution), insulin receptor (IR) (cat # mab 3025, Cell Signaling Technology, Inc, Danvers, MA; ~95 kDA; 1:500 dilution), SNAT1 (cat# MABN502, EMD Millipore, Temecula, CA; ~54 kDA, 1:500 dilution), SNAT2 (cat # sc-166366, Santa Cruz Biotechnology, Inc, Dallas, TX; ~60kDA; 1:500 dilution), and LAT2 antibody (cat# sc-27581, Santa Cruz Biotechnology Inc., Dallas, TX; ~58kDA; 1:1000 dilution). Anti-vinculin antibody (cat# V9131, Sigma-Aldrich, St. Louis, MO; ~117 kDA; 1:5000) was used to detect endogenous vinculin, which served as an internal control for inter-lane loading variability. The quantification of protein bands was performed by densitometry using ImageQuant software (GE Healthcare). The optical density was corrected for inter-lane loading variability using an internal control. For all bands, resultant optical density was ensured as linear to the loading protein concentrations, and the intensity of the protein bands was assessed by densitometry using the Scion Image software program [27].

#### 2.4 Dual-luciferase reporter studies

**2.4.1 Construction of the vector containing the reporter gene**: To construct a vector containing the complementary sequence to *miR-10b* fused to the 3’-end of the Renilla luciferase reporter, a dual luciferase psiCHECK^™^-2 vector (Promega, Madison, WI) was used. The sequence 5’ gccgctcgagtaccctgtagaaccgaatttgtggcggccgcgccg 3’ and its reverse sequence 5’ cggcgcggccgccacaaattcggttctacagggtactcgagcggc 3’ (Operon, Huntsville, AL) containing the enzymatic cleavage sites for XhoI and NotI were annealed and purified. Both the double stranded oligo and the psiCHECK^™^-2 vector were digested with XhoI and NotI restriction enzymes (New England BioLabs, Ipswich, MA). The oligonucleotide was then ligated within the vector using the Rapid DNA Ligation Kit (Roche, Indianapolis, IN) per the manufacturer’s instructions. A scrambled sequence 5’ gccgctcgagcctaactagacctaagcgcgaaagcggccgcgccg 3’ and its reverse sequence 5’ cggcgcggccgctttcgcgcttaggtctagttaggctcgagcggc 3’ (Operon) were created using the above methodology and served as negative controls. The orientation of the insert relative to the Renilla luciferase was confirmed by sequencing the construct using the primer: 5’ cgaggtccgaagactcattt 3’ (Bioneer, Alameda, CA). The basic vector psiCHECK^™^-2, which contains both the Renilla and Firefly luciferase reporter gene, served as an additional empty vector control.

**2.4.2 Transient Transfection of HTR8 cells with the construct**: HTR-8 cells were cultured and 2x10^5^ cells/well were seeded evenly in a 12-well culture plate to a final volume of 1 ml. The cells were incubated for 24 hours under standard conditions (95% air, 5% CO_2_, 37°C) and then transfected with either 200ng of empty psi-CHECK-2 vector, 200ng of the psi-CHECK-2/miR-10b construct, or 200ng of the scrambled construct using 3 μl of Lipofectamine^™^ 2000 (Invitrogen, Grand Island, NY) per manufacturer’s instructions. These studies were performed in quadruplicate.

**2.4.3 Luciferase Reporter Assay**: The luciferase activity was assessed using the Dual Luciferase Assay (Promega, Madison, WI). Briefly, 24 hours post-transfection the cells were washed with PBS and then lysed with 100 μl of 1X passive lysis buffer per manufacturer’s instructions (Promega, Madison, WI). The samples were stored at -70°C until analysis. A total of 10 μl of the cellular extract was analyzed using the Dual-Luciferase^®^ Reporter Assay kit (Promega) according to protocol. The luciferase activity was measured as light output (5s) using the Luminescence Microplate Reader (BioTek, Winooski, VT). Each vector construct was analyzed in duplicate. Activity of Renilla luciferase was normalized to Firefly luciferase, the latter serving as the transfection control.

### 3. MiRNA microarray and target gene pathway validation

For validation of target genes and pathways affected by differentially expressed miRs in IUGR versus AGA, seven AGA and six SGA/IUGR placental samples were selected and an attempt to minimize heterogeneity of the samples undertaken. Generally, pregnancies ending in cesarean delivery yielding a male offspring were chosen. miRNA was isolated as described in section 1.1, and subjected to genome-wide microarray analysis, by the Clinical Microarray Core facility at UCLA, using the more recent Affymetrix GeneChip miRNA 4.0 Array (Affymetrix, Santa Clara, CA). This array is far expanded from the previous version (3.0 Array) used for initial screening studies described in 1.2. The data obtained was initially analyzed using the Express Console^™^ Software version 1.4.1.46 (Affymetrix). For this study, similar to our initial screen, we focused on mature human miRNAs, labeled “hsa”. Once differentially expressed genes were detected (as defined in the “Statistical Analysis” section), the data set was re-analyzed. Target genes and associated KEGG pathways for miRs of interest were identified using miRWalk 2.0, where p-values (with correction for multiple testing methods, when applicable) <0.05 were considered significant [30].

### 4. Statistical analysis

For the initial screen microarray analysis, the expression values were estimated using Robust Multi-array Average (RMA). The empirical Bayes moderated t-test (EB) [31] was used to compare mean expression between AGA (n = 3) and SGA (n = 3) groups. EB p-values ≤0.05 were considered significant, resulting in 141 differentially expressed genes with a false discovery rate (FDR) of ~20%. MiRs were further filtered by focusing on microRNAs: (1) designated “hsa”, removing “U” and “ENSG” genes, and (2) with at least 2 present calls in either group (AGA or SGA). A heat map of the top 50 up-regulated candidates was constructed using agglomerative hierarchical clustering based on the average linkage function. Statistical analyses for the microarray analysis and heat map construction in both sets of experiments were carried out using R V3.1.2 (Vienna, Austria, www.r-project.org).

Up-regulated targets discovered in the initial microarray data and/or miRs of interest, based upon the literature related to their biological function were further investigated. *MiR*-*10b* and *363* were up-regulated in the microarray data and have been known to target genes mediating angiogenesis or nutrient transport. *MiR-149* was not found to be up-regulated in human IUGR placenta, but has been found previously in human placenta and circulation during pregnancy [32,33] and described to be up-regulated in placentas of an animal model of IUGR [18]. Genes were analyzed between AGA (n = 21) and SGA/IUGR (n = 11) groups using the Student’s t-test. Comparisons between more than 2 values were analyzed using one-way analysis of variance (ANOVA) with Fisher’s LSD. All statistical analyses were conducted in GraphPad Prism software (version 5, GraphPad Software Inc., La Jolla, CA). P-values <0.05 were considered statistically significant. Data were expressed as means ± SD, unless otherwise noted. Inter-group differences between two groups were determined by the Student t-test after confirming normal distribution. Significance was defined as p≤0.05.

For the expanded validation microarray analysis in section 3, the expression values were also estimated using Robust Multi-array Average (RMA). MiRs were filtered by focusing on microRNAs designated “hsa”, removing “U” and “ENSG” genes. The empirical Bayes moderated t-test (EB) [31] was used to compare mean expression between AGA (n = 7) and SGA (n = 6) groups. We found 28 miRs to be differentially expressed between the two groups, having both EB p-values <0.05 and a false discovery rate (FDR) <5%. The FDR adjustment procedure for both the pathway and miRNA analysis was computed using the Benjamini-Hochberg method [34]. A heat map of the 28 differentially expressed miRs was constructed by the agglomerative hierarchical clustering based on the average linkage function.

## Results

### Sample population characteristics

The maternal and infant characteristics related to the collected placentas are shown in Table 1. It should be noted that there were significantly more caesarian sections in the AGA group (76%) as compared to the SGA/IUGR group (27%) (p<0.02, by Chi-square testing). The associated clinical characteristics (gestational age at birth, sex of infant, mode of delivery, birth weight, length, head circumference, and notable prenatal ultrasound findings) of placentas collected are presented in S2 Table, with special notation of a sub-set of the IUGR placentas with accompanying ultrasound data showing fetal growth deceleration and/or abnormal umbilical artery or middle cerebral artery Doppler blood flow velocities.

**Table 1:** Maternal and infant characteristics.

	AGA (mean +/- SD)	SGA/IUGR (mean +/- SD)	p-value
**Number of samples**	21	10	
**Gestational age at birth (weeks)**	39.0 ± 1.0	38.7 ± 0.9	0.39
Birth weight (grams)*	3390.2 ± 435.1	2540.0 ± 204.7	< 0.0001
**Male**	11 (52%)	7 (64%)	0.35
Mode of delivery—Cesarean-section*	16 (76%)	3 (27%)	0.01

Differences between two groups validated with Student’s t-test or Chi-square testing, where appropriate.

* Significance assigned when p-value≤0.05.

### Genome-wide microarray

**Fig 1:**
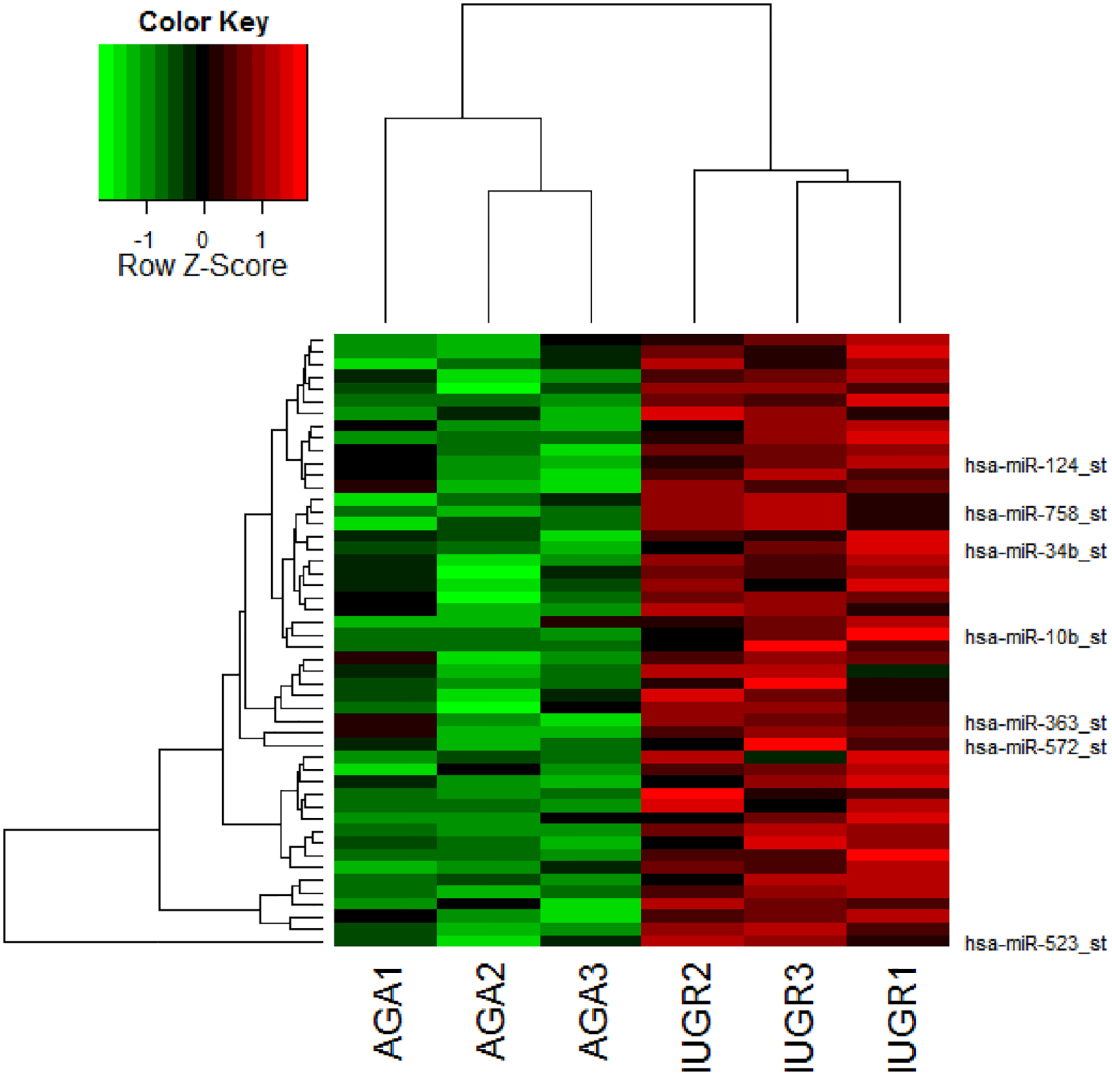
Heatmap of top miRNA candidates up-regulated in SGA/IUGR placentas compared to control placentas. Red indicates up-regulation and green indicates down-regulation of miRNA expression. This heatmap represents good differentiation of SGA/IUGR from AGA (individual samples listed across the x-axis) based upon the top 50 miRNAs identified as up-regulated in the SGA/IUGR group by microarray (depicted along the y-axis). Selected top candidates, including *miR-10b* and *-363* are called out to the right of the heatmap.

Based on our criteria for significance, there were 97 up-regulated and 44 down-regulated miRNAs in the SGA group as compared to the AGA with an EB p-value ≤ 0.05 (S3 Table). The heatmap constructed (Fig 1) of up-regulated genes in the SGA group shows good discrimination between the AGA and IUGR groups, despite small sample sizes and variability in clinical characteristics such as mode of delivery and sex of the infant, as does the heatmap of down-regulated genes (S1 Fig). All samples were carefully selected for full term singleton pregnancies, non-complicated by maternal health conditions such as infection, which was necessary in reducing inter-sample variability. We observed that most of the up-regulated miRs regulated genes relevant to hypoxia responses [35–39], vascular disease [40–44], metabolism [45–49], inflammation [50–51], or were previously reported in pre-eclamptic placentas [52–55]. In contrast, the down-regulated miRs were for the most part relevant for genes with a role in obesity or cancers [56–58], though several had also been reported in association with pregnancy disorders involving placental insufficiency [52, 55,59–61]. Given our focus on up-regulated miRs with the potential of down-regulating target genes, selected genes in the top 50 miRs which were differentially expressed (up-regulated) between groups are listed to the right of the figure, including *miR-10b* and *-363*, which were further validated in subsequent studies.

We chose to focus further investigations on *miR-10b*, *miR-363*, and *miR-149* based on the following criteria: a) mean signal values greater than background, b) established significance of these miRNAs in placental function by others and our own murine studies [18,32,33], and/or c) target mRNAs with known function pertinent to angiogenesis, nutrient transfer, cell proliferation and/or patterning/development. We chose these functions that are critical for placental health necessary in ensuring fetal growth.

All microarray data are publically available in the GEO database, under accession number GSE93174.

### Validation of microarray results by RT-qPCR

**Fig 2:**
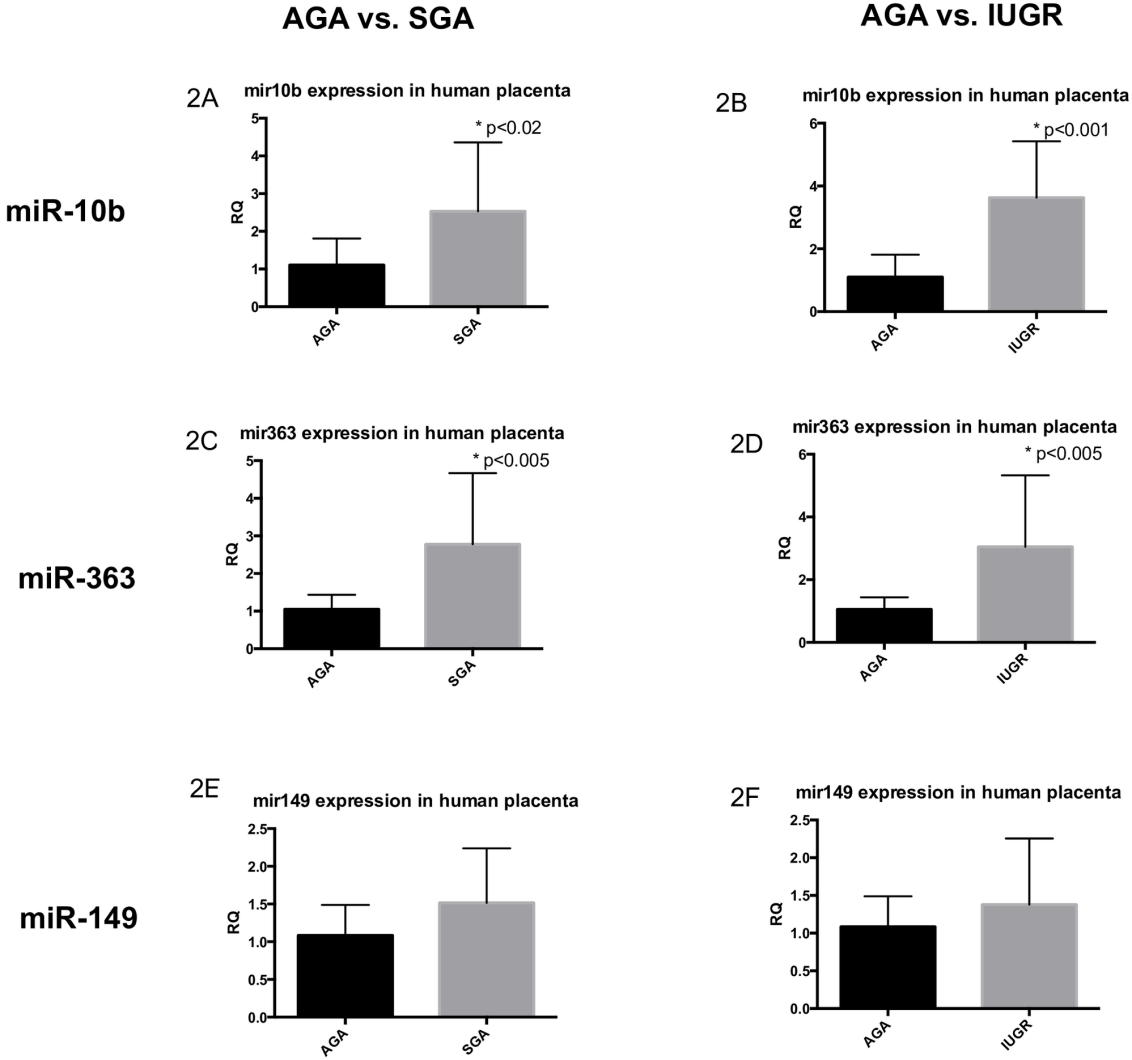
qRT-PCR validation of miRs of interest in human placental samples. The left column represents comparison of AGA to SGA samples, and the right column represents comparison of AGA to IUGR samples. The first row depicts data for *miR-10b* (Panels 2A, 2B), the second row for *miR-363* (Panels 2C, 2D), and the third row for *miR-149* (Panels 2E, 2F). All data are represented as means ± SD. Asterisks indicate significance, with p-values<0.05 by Student’s t-test.

Given that a small cohort of samples was used for microarray analyses, we validated miR expression in larger AGA versus SGA and IUGR cohorts employing RT-qPCR. Using the comparative Ct method, *miR-10b* had a 2.2 fold increase in expression in the SGA placentas as compared to the AGA (SGA: 2.5332±1.8289 versus AGA: 1.1045±0.7057; p-value≤0.02 by Student’s t-test) (Fig 2A). When only the IUGR subset was analyzed separately, this fold change was even greater, at 3.3-fold (IUGR subset: 3.6275±1.7926 versus AGA: 1.1045±0.7057; p-value≤0.001 by Student’s t-test) (Fig 2B). There was a 2.6-fold increase in *miR-363* in SGA placentas as compared to AGA (SGA: 2.7768±1.8919 versus AGA: 1.0507±0.3861; p-value≤0.005 by Student’s t-test) (Fig 2C). Again, when only the sub-set of IUGR samples was considered, this fold change was even greater, at 2.9-fold (IUGR subset: 3.0465±1.0193 versus AGA: 1.0507±0.3861; p≤0.005 by Student’s t-test) (Fig 2D). Consistent with the directional up-regulation seen in microarray (but did not reach statistical significance), *miRNA-149* expression was not different between SGA/IUGR and AGA placentas (AGA: 1.2770±0.7339 versus SGA: 1.4885±0.6284 versus IUGR subset: 1.4942±0.8055) (Fig 2E and 2F).

Western blotting of downstream targets KLF4 and LAT2 in human placenta did not reveal significant changes (data not shown). We believe that changes in target expression could not be detected because whole human placental samples have heterogeneous cell populations. Therefore, we undertook in-vitro investigations with pure trophoblast cells.

### In-vitro nutrient restriction experiments

**Fig 3:**
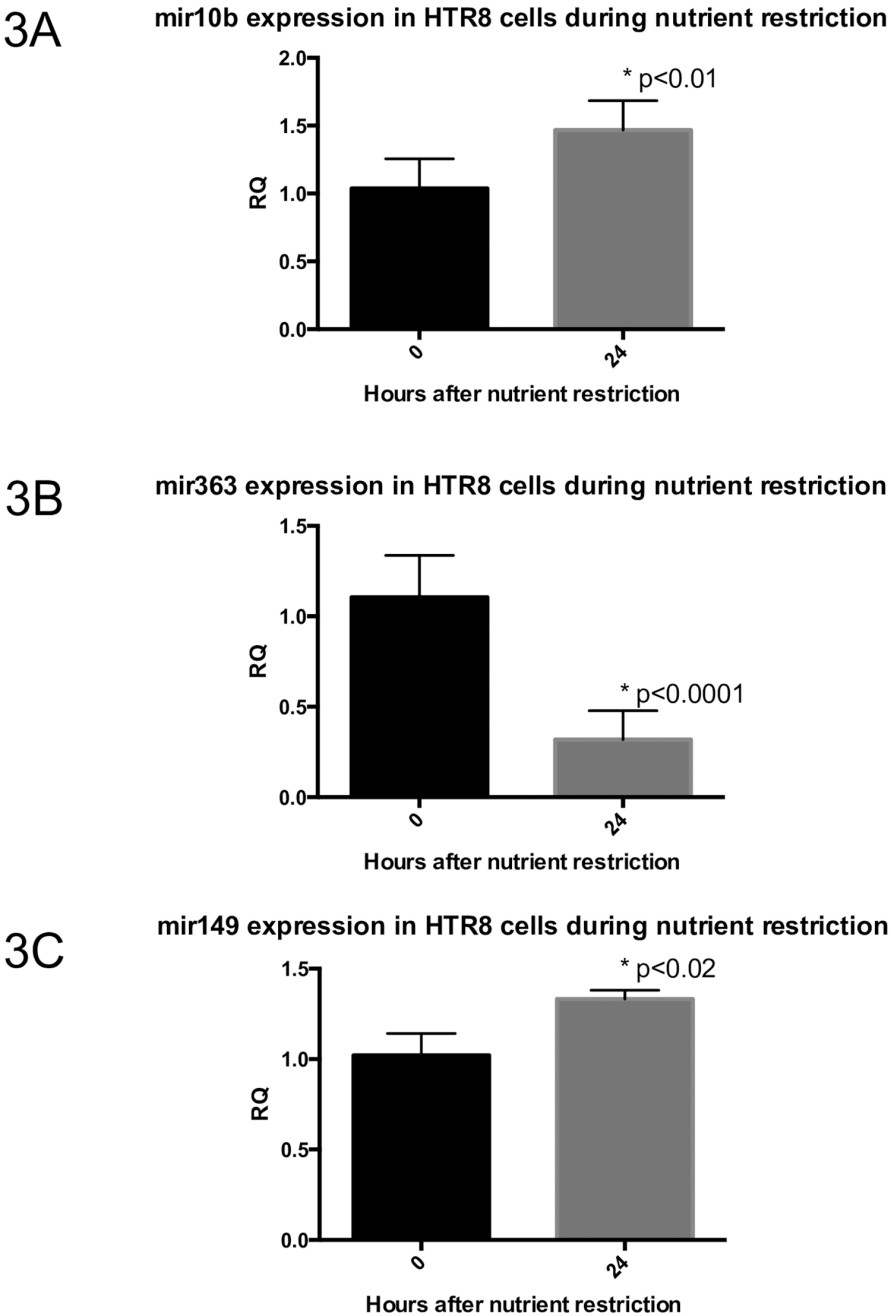
miR expression after 24 hours of 50% nutrient restriction in HTR8 trophoblast cells. (3A) *miR-10b* expression increases after 24 hours of NR, (3B) *miR-363* expression decreases after 24 hours of NR, and (3C) *mir-149* expression increases after 24 hours of NR in HTR8 trophoblast cells, but does not achieve statistical significant (p<0.07). All data are expressed as means ± SD. Asterisks denote significance, with p-value<0.05 by Student’s t-test for comparison of 24hr level expression to baseline expression.

In order to partially replicate the in-utero environment experienced by the human IUGR we next focused on two trophoblastic cell lines. HTR8 trophoblast cells were exposed to diluted media solutions to mimic nutrient restriction (NR). 50% dilution was chosen to mimic murine models of IUGR, in which 50% food restriction resulted in uteroplacental insufficiency and IUGR, and in which *miRs-10b* and *149* have been shown to be differentially expressed [18]. To assess whether *in vitro* nutrient restriction affected miRNA expression in trophoblast cells, we collected cells and performed RT-qPCR to quantify expression of microRNAs of interest under control baseline conditions, and also after 1 hour, 3 hours, 6 hours, and 24 hours of nutrient restriction. For all miRs of interest (-10b, -363, -149), gene expression was significantly changed at 24 hours of NR compared to baseline, by Student’s t-test (p<0.02, all) (Fig 3A–3C). We found that *miR-10b* expression was increased after nutrient restriction, compared to baseline expression under control conditions (baseline: 1.0382±0.2179 versus 1 hour post-NR: 1.1961±0.2135 versus 3 hours post-HR: 1.0181±0.3613 versus 6 hours: 1.6248±0.1695 versus 24 hours post-NR: 1.4670±0.2171; p<0.001 by ANOVA and Fisher PLSD). *miR-363* was also detected under control conditions and interestingly, expression was significantly decreased following nutrient restriction (baseline: 1.1052±0.2323 versus 1 hour post-NR: 1.0378±0.3625 versus 3 hours post-NR: 0.7080±0.3130 versus 6 hours post-NR: 1.1045±0.2218 versus 24 hours post-NR: 0.3176±0.1601; p<0.0001 by ANOVA with Fisher PLSD). This was opposite to what was seen in whole placentas exemplifying differences between pure trophoblasts versus a mixture of cell types. Although *miR-149* was no different in human IUGR placentas versus the AGA counterparts, in-vitro in pure trophoblast cell cultures, *miR-149* expression was increased at 24 hours following nutrient restriction, but did not reach significance when all time points were simultaneously compared (baseline: 1.0206±0.1211 versus 1-hour post-NR: 1.0143±0.2223 versus 3-hour post-NR: 1.0011±0.1331 versus 6 hours post-NR: 1.1129±0.0723 versus 24 hours post-NR: 1.3322±0.0490; p<0.07 by ANOVA). All time points for each miRNA of interest are shown in S2 Fig

Given differences noted between whole placenta and pure trophoblast cell cultures, we sought to determine the effect on targets by inhibiting endogenous miRs.

### Inhibition of endogenous miRs

**Fig 4:**
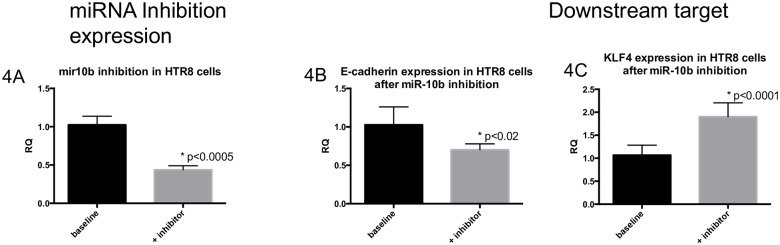
Inhibition of *miR-10b* in HTR8 cells affects expression of targets *E-cadherin* and *KLF4*. (4A) *miR-10b* expression levels in HTR8 cells at baseline control conditions, and after transfection with inhibitor. (4B) *E-cadherin* gene expression by qRT-PCR decreases after inhibition of *miR-10b* in HTR8 cells, compared to control conditions. (4C) *KLF4* gene expression by qRT-PCR increases after inhibition of *miR-10b* in HTR8 cells. All data expressed as means ± SD. Asterisks indicate significance with p-value<0.05, by Student’s t-test.

Cell cultures of HTR8 and BeWo were treated with the specific miRNA inhibitors and expression levels of downstream targets for the microRNA of interest were evaluated using qRT-PCR or Western blotting. For *miRNA-10b*, gene and protein expression of downstream targets, *E-cadherin*, *HoxD* and *KLF-4*, were assessed after inhibition of *miRNA-10b*. Inhibition of *miR-10b* was successfully achieved, as validated by qRT-PCR assessment of endogenous *miR-10b* expression in our negative control versus inhibitor conditions (control: 1.0239±0.1136 versus inhibition: 0.4365±0.0535; p<0.0005 by Student’s t-test) (Fig 4A). Gene expression of downstream targets *E-cadherin* was significantly decreased after *miRNA-10b* inhibition (control: 1.0269±0.2331 versus inhibition: 0.7023±0.0777; p<0.05 by Student’s t-test) (Fig 4B) and *KLF4* gene expression was significantly increased (control: 1.0677±0.2170 versus inhibition: 1.9030±0.3021; p<0.0001 by Student’s t-test) (Fig 4C). Gene expression of *HoxD10* did not change, and protein expression of E-cadherin, HoxD10 and VEGF were not significantly changed after inhibition (negative data not shown).

**Fig 5:**
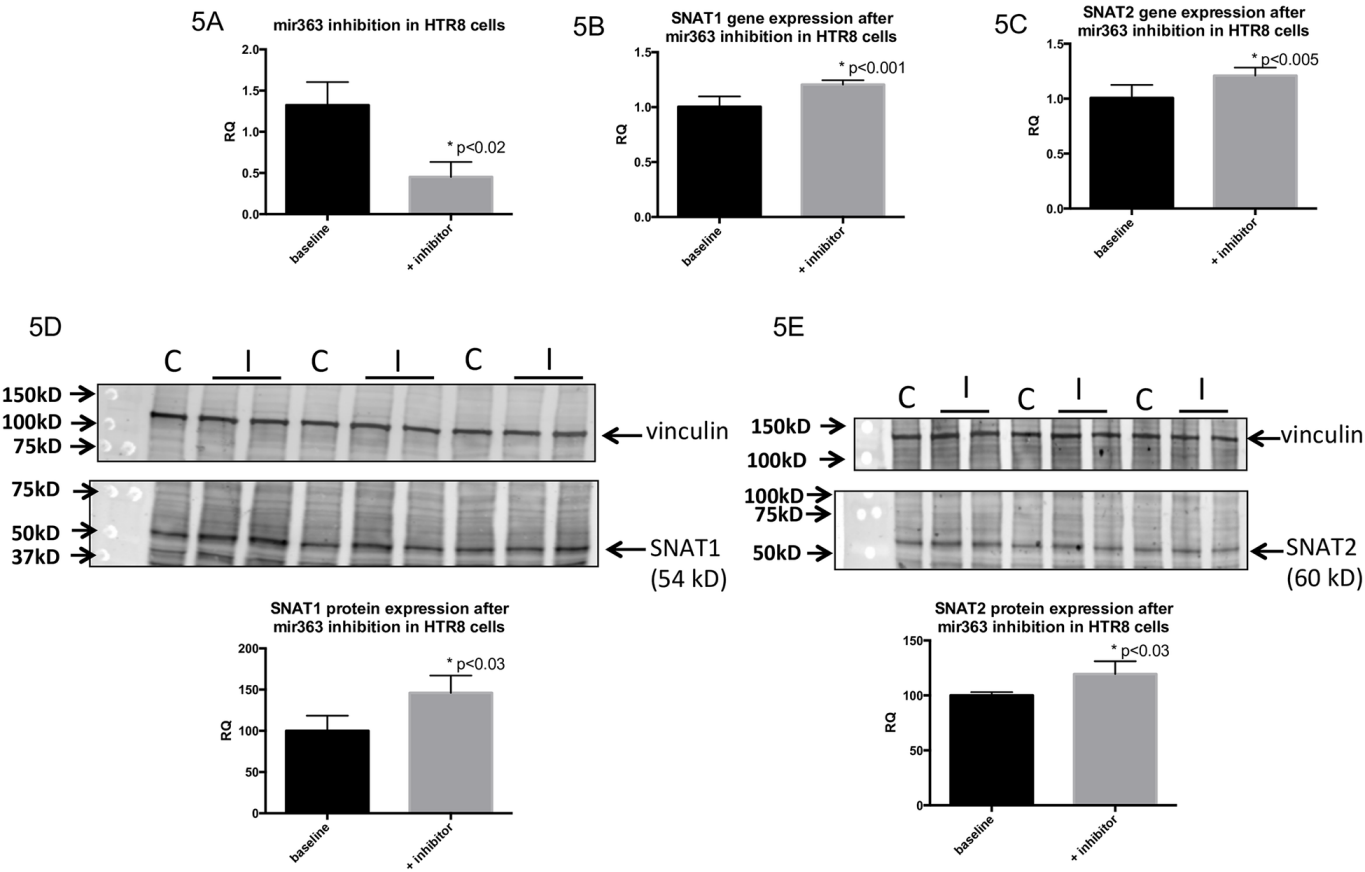
Inhibition of *miR-363* in HTR8 cells results in increased amino acid transporter expression. (5A) *miR-363* expression levels in HTR8 cells at baseline control conditions and after transfection with inhibitor. (5B) *SNAT1* gene expression by qRT-PCR increases after inhibition of *miR-363* in HTR8 cells, compared to at control conditions, as does *SNAT2* (5C). Protein levels of SNAT1 (5D) and SNAT2 (5E) in HTR8 cells as measured by western immunoblotting also increase after inhibition of *miR-363*. All data expressed as means ± SD. Asterisks indicate significance with p-value<0.05, by Student’s t-test.

For *miRNA-363*, inhibition of gene expression was confirmed (control: 1.3242±0.2791 versus inhibition: 0.4527±0.1812; p<0.02 by Student’s t-test) (Fig 5A). Gene expression of downstream targets of *miRNA-363*, *EGF*, *Glut-3*, *insulin receptor*, *SNAT1* and *SNAT2*, were evaluated by qRT-PCR. Only *SNAT1* and *SNAT2* demonstrated significant increase in gene expression after *miRNA-363* inhibition (*SNAT1* mRNA: control: 1.0045±0.0935 versus inhibition: 1.2053±0.0406; p<0.001) (Fig 5B) (*SNAT2* mRNA: control: 1.0066±0.1188 versus inhibition: 1.2109±0.0728; p<0.005 by Student’s t-test) (Fig 5C). Protein expression of *SNAT1* and *SNAT2* was also significantly increased after *miR-363* inhibition (SNAT1 protein: control: 100±18.47 versus inhibition: 146.0022±21.10; p<0.03 by Student’s t-test) (Fig 5D) (SNAT2 protein: control: 100±2.969 versus inhibition: 119.394±11.65; p<0.03 by Student’s t-test) (Fig 5E). The other targets tested failed to demonstrate a change (negative data not shown).

**Fig 6:**
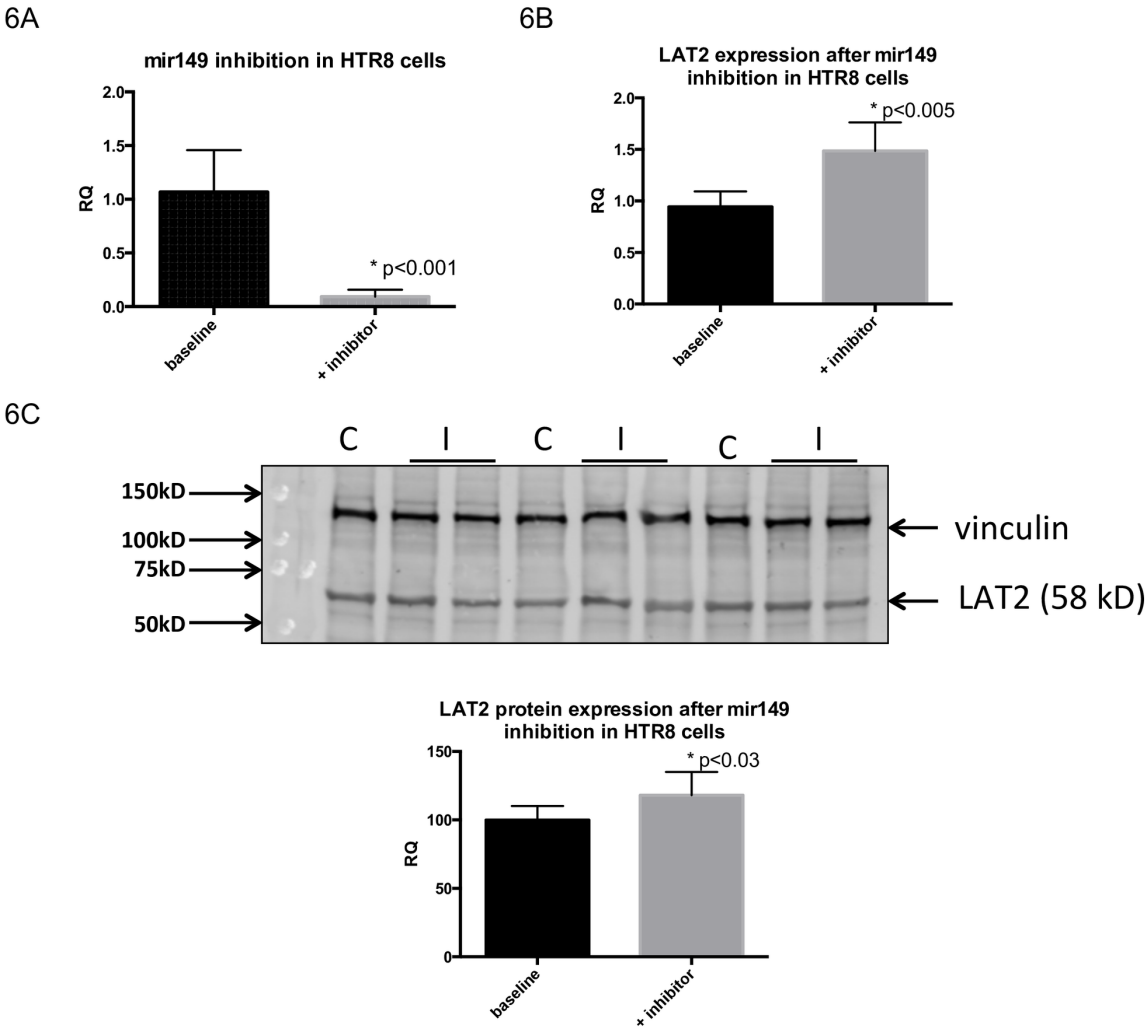
Inhibition of *miR-149* in HTR8 cells increases gene and protein expression of target gene *LAT2*. (6A*) miR-149* expression levels in HTR8 cells at baseline control conditions and after transfection with inhibitor. (6B) *LAT2* gene expression by qRT-PCR increases after inhibition of miR-149 in HTR8 cells, compared to control conditions, as does protein expression of LAT2 (6C). All data expressed as means ± SD. Asterisks indicate significance with p-value<0.05, by Student’s t-test.

For *miR-149*, *LAT2* expression was evaluated by both qRT-PCR and western blotting. *miR-149* inhibition was successful (control: 1.0674±0.3897 versus inhibition: 0.0931±0.0639; p<0.01) (Fig 6A), and concomitant *LAT2* gene expression was significantly increased after *miR-149* inhibition (control: 0.9424±0.1509 versus inhibition: 1.4855±0.2764; p-value<0.005) (Fig 6B), as was LAT2 protein expression (control: 99.9999±10.22 versus inhibition: 118.0610±16.96; p<0.03 by Student’s t-test) (Fig 6C).

Employing the BeWo cell line that exhibits trophoblast cell syncytial characteristics, we observed successful inhibition of *miR-10b* and *-363* similar to the HTR8 cell line, although *miR-149* unlike the HTR8 cells failed inhibition. Unlike HTR8 cells, despite successful suppression of *miR-10b* and *-363*, no change in the different targets tested was observed (data not shown). These results attest to fundamental differences in cancerous cell lines (BeWo) from transformed innate trophoblast cells (HTR8), with the cancer cells exhibiting escape from normal post-transcriptional regulatory checks imposed in trophoblast cells.

### *In vitro* functionality using dual-luciferase assays

**Fig 7:**
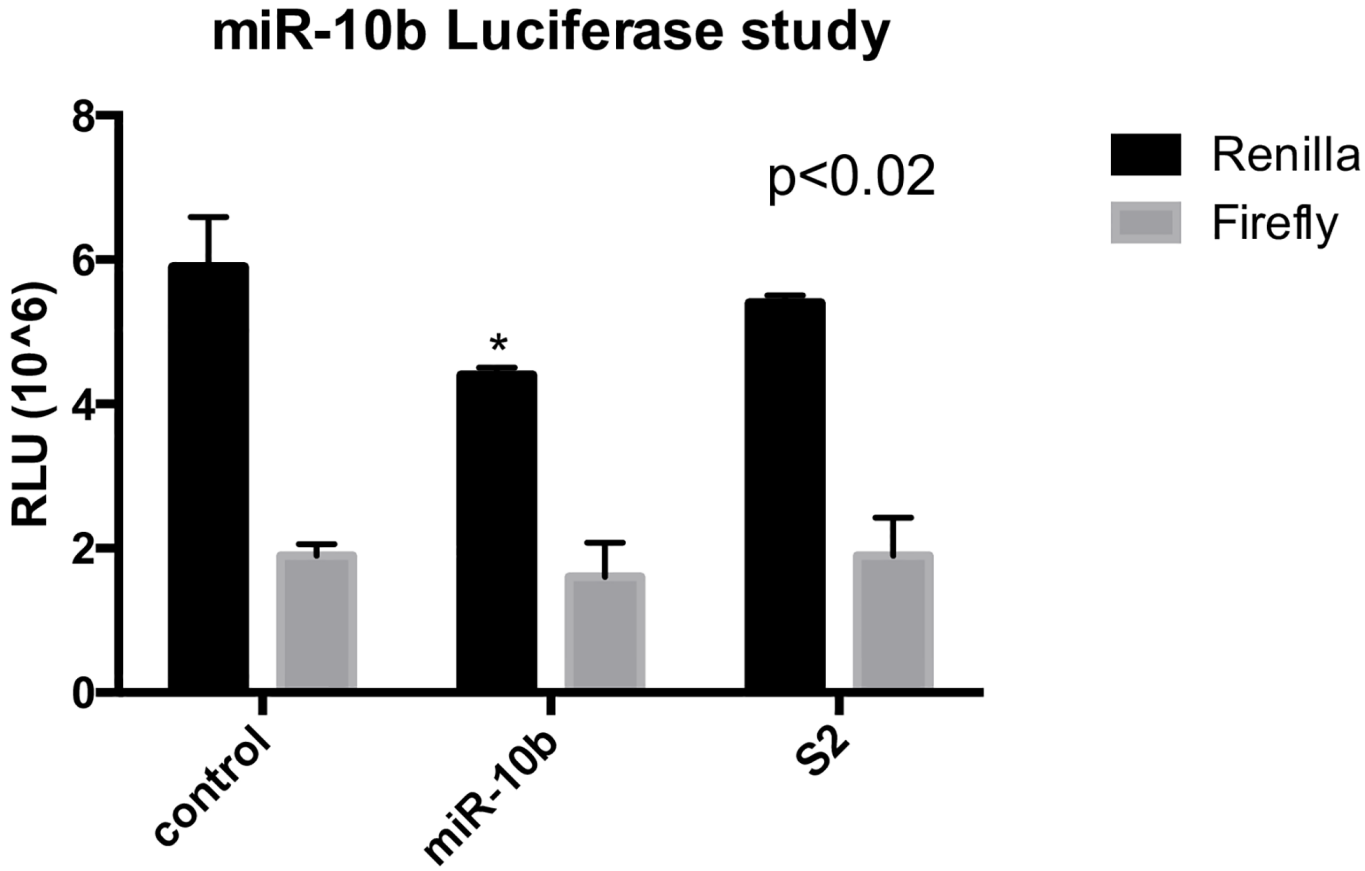
*MiR-10b* functionally binds its complementary mRNA sequence in HTR8 cells, as measured by the dual-luciferase assay. Renilla expression is decreased with miR-10b vector, as compared to negative control and scrambled sequence expression. Firefly expression is not different between groups, indicating similar transfection efficiency. All data expressed as means ± SD. Asterisks indicate significance with p-value<0.05, by Student’s t-test.

To confirm that miRNAs of interest expressed in trophoblasts do bind to their complementary sequences present on target mRNAs and suppress subsequent mRNA translation, the complementary sequences were cloned into the psi-CHECK-2 vector downstream of the Renilla luciferase reporter. A scrambled insert was also created and inserted into a psi-CHECK-2 vector to serve as a negative control. After transfection of both constructs and an empty vector into HTR-8 cells, the relative Renilla luciferase expression of the vector containing the complementary *miR-10b* sequence was significantly decreased (p≤0.001) compared to the empty vector and the scrambled sequence (control: Renilla 5.9±0.69, Firefly 1.9±0.16; miR-10b: Renilla 4.4±0.1, Firefly 1.6±0.48; S2: Renilla 5.4±0.1, Firefly 1.9±0.52; p≤0.01) (Fig 7). The transfection efficiency was similar between the three vectors as indicated by no change in the Firefly luciferase activity. This indicates a direct and specific interaction of the endogenous *miR-10b* with its complementary mRNA sequence associated with a ~10% reduction in the translation of the 5’-luciferase gene expression into biological activity of light emission, thereby assigning a biological function to this miR, as a proof of principle.

### Validation of microarray results

**Fig 8:**
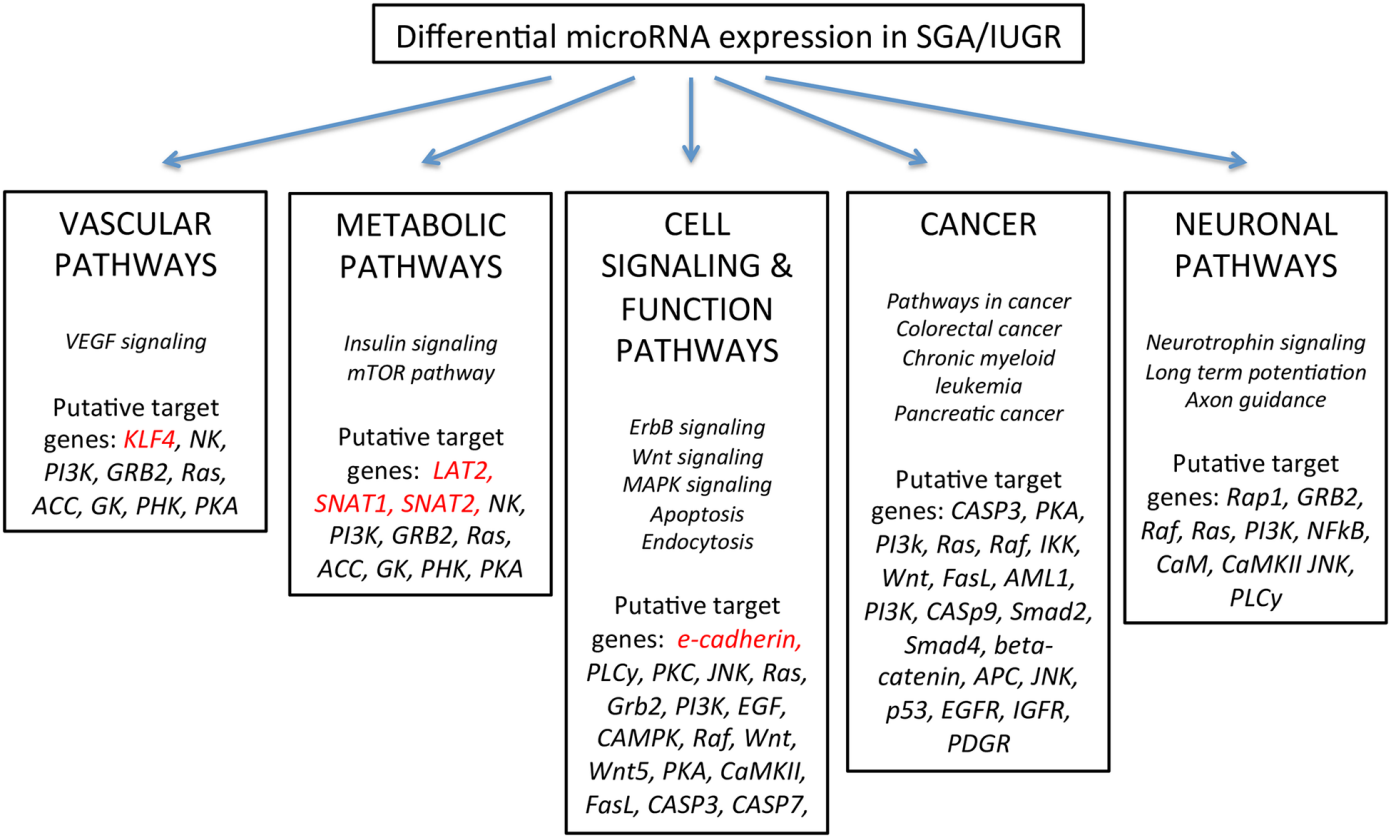
Predicted significant pathways on putative target genes of differentially expressed miRNAs in SGA versus AGA placenta. Predicted pathways on putative target genes (3-UTR region) of top differentially expressed miRNAs in SGA versus AGA placentas. Both the initial screening and subsequent expanded validation microarray sets consistently had these same pathways (in italics) highly represented among the differentially expressed miRs. We also highlight target genes of interest (e-cadherin, KLF4, LAT2, SNAT1, and SNAT2) validated in our studies, as well as target genes identified by pathway analyses.

Given the initial small sample sizes used to screen for candidate miRs and target genes, we performed repeat microarray on a larger cohort of subjects (n = 6–7 per AGA and SGA groups) to validate that our specific target genes of interest are reliable and replicable in a different cohort. We specifically limited covariates (sex of neonate, and mode of delivery) in order to minimize sample heterogeneity. We found 28 microRNAs that were differentially expressed in SGA/IUGR versus AGA placentas (S3 Fig). Though the specific miRs that were identified as differentially expressed initially (version 3.0) were different now (version 4.0), all five of our target genes of interest (CDH1/e-cadherin, KLF4, LAT2, SLC38A1/SNAT 1, and SLC38A2/SNAT2) were still identified as target genes among the 28 differentially expressed miRs in SGA versus AGA placentas (Table 2). These additional differentially expressed miRs between SGA/IUGR versus AGA placentas will need future confirmation by qRT-PCR in a separate study. Pathway analyses performed on both the initial screening microarray subset and this validation subset however showed consistent patterns in the top KEGG pathways represented by the differentially expressed miRs (Fig 8).

**Table 2:** Differentially expressed miRs in AGA versus SGA placentas in validation microarray.

Transcript ID	Average expression of AGA samples	Average expression of SGA samples	p-value (by modified EB testing	FDR	Target gene of interest (p-value)*
miR-202-3p	2.30	3.79	p<0.0001	0.008	
miR-3921	6.72	5.75	p<0.0001	0.018	LAT2 (p = 0.02)
miR-525-3p	13.03	12.29	p<0.0001	0.020	
miR-4428	3.14	4.53	p<0.0001	0.020	
miR-5703	2.64	4.00	p<0.0001	0.020	
miR-525	11.45	10.66	p<0.0001	0.020	
miR-5002	2.60	1.81	p<0.0001	0.020	
miR-4419a	2.23	3.41	p<0.0001	0.021	SLC38A1 (p = 0.0056)
miR-525	11.45	10.69	p<0.0001	0.021	
miR-6880	6.64	5.41	p<0.0001	0.021	
miR-520f-5p	6.41	5.41	p<0.0001	0.028	
miR-4773	2.03	3.16	p<0.0001	0.031	
miR-1225	3.62	2.86	p<0.0001	0.031	CDH1 (p = 0.03)
miR-6857	3.41	2.42	p<0.0001	0.031	
miR-6739-5p	1.80	2.72	p<0.0001	0.043	SLC38A1 (p = 0.006)
miR-518f-5p	11.39	10.74	p<0.0001	0.046	
miR-526a	11.75	11.12	p<0.0001	0.046	
miR-520c-5p	11.75	11.12	p<0.0001	0.046	
miR-519d-5p	8.23	7.18	p<0.0001	0.046	
miR-518d-5p	11.75	11.12	p<0.0001	0.046	
miR-3125	1.55	2.14	p<0.0001	0.046	SLC38A1 (p = 0.0056)
miR-3910	2.38	3.78	p<0.0001	0.046	
miR-548y	2.59	1.95	p<0.0001	0.046	KLF4 (p = 0.013), SLC38A2 (p = 0.04)
miR-4506	2.30	3.44	p<0.0001	0.046	
miR-4514	2.05	2.93	p<0.0001	0.046	
miR-6821-3p	2.23	3.10	p<0.0001	0.046	
miR-6845-5p	4.19	5.32	p<0.0001	0.046	SLC38A1 (p = 0.02)

* Significance assigned when p-value<0.05. SLC38A1 = SNAT1, CDH1 = e-cadherin, SLC38A2 = SNAT2.

All validation microarray data are publically available in the GEO database, under accession number GSE97294.

## Discussion

We have found that human IUGR is associated with a distinct microtranscriptomic signature, as has been reported in other pathologic pregnancy complications such as pre-term birth, pre-eclampsia, and gestational diabetes [21–24,62–64]. By miRNA microarray, we discovered 141 candidate miRNAs that are differentially expressed in the placenta associated with a cohort of SGA and IUGR neonates compared to their AGA counterparts. We selected candidate micro-RNAs up-regulated in IUGR with known function in vascular development, nutrient transfer, or cell proliferation, as these processes are essential for optimal placental formation and function to provide for the growing fetus, and validated their differential expression in a larger cohort of IUGR placentas compared to AGA-associated placentas. To establish a cause-and-effect paradigm, using a model of nutrient restriction of trophoblast cells *in vitro* to mimic human and an animal model of IUGR secondary to nutrient restriction resulting in placental insufficiency, we demonstrate that nutrient deprivation to trophoblasts alters the expression of our candidate miRNAs, e.g. *miR-10b*, *miR-363*, *and miR-149*. We then evaluated whether inhibition of these miRNAs affected the gene and protein expression of their downstream targets, thereby establishing their biological function. In addition, we evaluated whether one of these miRNAs, namely *miR-10b* also demonstrates specific binding to their complementary sequences *in vitro* using luciferase reporter assays, attempting to further delineate the molecular impact of such 3’-UTR sequence complementation on post-transcriptional processing of genes within trophoblast cells.

The first candidate miRNA that we evaluated for functional importance in trophoblast cells was *miR-10b*. Its expression was significantly increased in IUGR by microarray and by qRT-PCR validation. *miR-10b* is located within the homeobox cluster of genes located on chromosome 17 and is therefore co-regulated with the Hox genes and affects cellular migration and invasion. One particular target gene of interest is *E-cadherin*, which belongs to a class of type-1 transmembrane proteins important in cellular adhesion. Liu, et al. showed that *miR-10b* regulates the expression of *E-cadherin* in a breast cancer cell line [65]. Further, down-regulation of *E-cadherin* leads to decreased strength of cellular adhesions and increased cellular motility. Similar to the proposed mechanism for *HOXD10*, the down-regulation of *E-cadherin* by *miR-10b* in cases of fetal growth restriction could serve as a pathway for these placentas to mitigate the effects of uteroplacental insufficiency. Our in-vitro studies in trophoblasts demonstrated a down-regulation of *E-cadherin* upon inhibition of endogenous *miR-10b*, supporting intermediaries responsible for this effect. If extrapolated to the in-vivo situation, increased placental *miR-10* in IUGR placentas may reflect increased *E-cadherin* ensuring maturation of cell adhesions towards premature trophoblastic terminal differentiation rather than proliferation, underlying the small sized placentas. On the other hand, *KLF4*, a nuclear transcription factor, has been shown to play a number of regulatory roles, including in immune activation, cell growth, and in particular, angiogenesis [66–68]. Inhibition of *miR-10b* led to up-regulation of *KLF4 in vitro* suggesting that *KLF4* suppression as a result of up-regulation of *miR-10b* in human IUGR placentas in-vivo. This may lead to impaired angiogenesis or alteration of immunologic environment at the maternal-fetal interface, both processes that have been described in association with IUGR. Quantification of human placental *KLF4* may not reflect the changes observed in-vitro, due to multiple cell types in the human placenta beyond trophoblasts and the fact that *KLF4* is a nuclear protein, its biological activity being displayed as transcriptional regulation upon DNA-binding of downstream genes.

*miRNA-363* was also up-regulated in the initial microarray data, and validated by the whole placental qRT-PCR data. Interestingly, nutrient restriction *in vitro* of trophoblast cells resulted in lowering of *miR-363* expression. While its role in human disease is not well described, down-regulation of *miRNA-363* in pre-eclamptic placentas has been described [52,69]. This discrepancy between whole IUGR placentas and in-vitro nutrient restriction (~50%) upon *miR-363* expression may relate to the lack of ischemia in-vitro that is the end result of utero-placental insufficiency in-vivo. Further, when examining whole placenta, cells beyond trophoblasts alone are included, however in-vitro studies consist of trophoblast cells exclusively. In addition, the trophoblast cell lines were either transformed or cancerous and may not completely mimic placental trophoblasts in-vivo. Nevertheless, to establish a cause-and-effect paradigm it was necessary to employ such trophoblast cell lines that could accommodate transient transfection of exogenous genes (unlike primary cultures of human trophoblast cells) for our subsequent studies. Most importantly, our present in-vitro studies demonstrated a change in *miR-363* in response to nutrient restriction alone. Targets of *miR-363* include *SNAT1*, *SNAT2*, insulin receptor, epidermal growth factor, and glucose transporter 3 (G*LUT3*). Of those targets, only *SNAT1* and *SNAT2* demonstrated differential gene and protein expression in trophoblasts after *miR-363* inhibition. *SNAT1* and *SNAT2* are sodium coupled neutral amino acid transporters, whose perturbed expression may affect overall trophoblastic uptake of small non-essential neutral amino acids such as alanine, glycine and serine [70]. This transporter system enables uptake of amino acids against their concentration gradient by simultaneously transporting sodium into the cell. Importantly, system A activity contributes to the high intracellular concentrations of amino acids such as glycine, which are exchanged for extracellular essential amino acids transported by another system L amino acid transport system. System A is thus important for placental transport of both non-essential and essential amino acids. In our studies, the reciprocal effect of *miR-363* on *SNAT1* and *SNAT2* mRNAs and protein sets the stage for extrapolation to the in-vivo situation of human IUGR placentas. Again in the IUGR placenta, *miR-363* increased in our present study, and as demonstrated by others, the IUGR placenta revealed reduced *SNAT 1* and *SNAT2* concentrations and System A amino acid transport activity [70–73].

We also evaluated *miRNA-149* in our human placentas and *in vitro* studies. Expression of *miRNA-149* did not change in the studied IUGR placentas, or in the microarray results. Previous studies employing IUGR and/or pre-eclamptic placentas demonstrated controversial results, with one study demonstrating no significant change with IUGR, while another displayed a reduction in placental *miR-149* with pre-eclampsia [32]. Of intrigue was the observation that *miR-149* was detected in the maternal circulation as long as gestation lasted increasing with advancing pregnancy, only to precipitously drop upon parturition [33]. Despite there being no change in our present human IUGR placental samples, we chose to study this miRNA in-vitro to establish the reason for its presence in human placenta and to determine its functional relevance to gestation.

Our previous investigations in the IUGR murine model demonstrated up-regulation of *miR-149* [18] in placentas, which was not seen in our present human samples. However, nutrient restriction in-vitro mimicked our previous in-vivo calorie restricted IUGR murine placental observations [18], where an increase in *miR-149* expression was observed. Further, in-vitro knock down of *miR-149* increased its target *LAT2* expression. *LAT2* is a system L transporter isoform localized to the basal membrane of the syncytiotrophoblastic cellular layer, and is a sodium independent obligatory exchanger of neutral amino acids. Non-essential amino acids are exchanged for predominantly essential amino acids, with either aromatic or branched side chains (e.g. leucine and phenylalanine) enabling transport against their concentration gradient. The basal membrane localization of *LAT2* allows for exchange of amino acids between non-essential amino acids in the fetal compartment with essential amino acids in the cellular cytoplasm. Thus *LAT2* plays a key role in mediating net efflux of essential amino acids to the developing fetus, and may be affected in human IUGR placentas [74–76]. In particular branched chain amino acids play a major role in fetal growth and are diminished in IUGR fetuses [77]. All these studies cumulatively suggest that a more severe state of human IUGR may invoke higher *miR-149* expression and thereby its down regulation of *LAT2* mediated branched chain amino acid transfer to the fetus.

Generally miRs demonstrate low fidelity to their target mRNAs providing a basis for their influence upon a network of genes responsible for a particular biological process or associated pathways. Acknowledging this property, we evaluated only a few target genes found via computer-based search databases for each candidate miR identified and thoroughly validated in our study. We presented only specific targets that demonstrated a change and were concerned with angiogenesis and nutrient transfer. However, we subsequently undertook an expanded microarray analysis in a larger, more homogenous population of SGA and AGA placentas to demonstrate reliability and replicability of our results. The validation microarray detected many more differentially expressed miRs. Most importantly, the screening (n = 3 each) and the subsequent expanded (n = 6–7 each) microarray analyses both demonstrated all five of our target genes of interest (e-cadherin, KLF4, LAT2, SNAT1, and SNAT2). In addition, pathway analyses confirmed that in both cohorts (the smaller initial cohort used for screening and the larger expanded cohort used for validation), the same pathways are predicated on putative target genes for differentially expressed miRs. Among these pathways, vascular, metabolic, and cellular integrity pathways were highly represented and are biologically important both for fetal growth but also for risk prediction of future disease. In addition, our microarray data provides insight into additional potentially important pathways affected by differential miRNA expression in intrauterine growth restriction.

Thus, our study demonstrated the biological significance on a cause-and-effect basis of certain identified placental miRs that were perturbed in the condition of human late gestation IUGR. Though our microarray studies performed on a subset of patients also demonstrated down-regulation of miRs that have been previously shown to be differentially expressed in conditions associated with placental insufficiency and adverse in utero environmental exposures (e.g. *miRs-519a*, *518e*, *21*, *183*, *519c*, *522*, *523*, *99b*) [52, 55, 59–61], we did not pursue further studies on these genes as there has not been identified functional implications of these miRs with angiogenesis or nutrient transfer. However, it was intriguing to note that several of the down-regulated miRs identified via microarray are associated with overgrowth syndromes, such as obesity, lipid deposition, and cancer [56–58]. Future investigations will identify and study other miRs identified from our combined microarray studies and other targets identified by the TargetScan/miRWalk analysis towards demonstrating a network of genes responsible for aberrant placental morphology which perhaps leads to fetal growth restriction (IUGR).

In summary, *microRNAs -10b* and *-363* are up-regulated in the human term IUGR placenta. These micro-RNAs have not been well described in placental pathology, and are not found in the known placental specific miRNA clusters. Further, *miR-10b* and *-363* are likely superior markers of true fetal growth restriction given the exaggeration in fold-change seen when evaluating only those IUGR samples with ultrasound evidence of fetal growth deceleration and compromise. Using nutrient restriction as a surrogate for intrauterine stress, *miR-10b* was shown to increase and *-363* was shown to decrease following a dilution in cell culture media indicating altered regulation in response to nutrient restriction which is a major biological contributor in uteroplacental insufficiency. Inhibition of these miRNAs led to alterations in the gene and protein expression of some of their downstream targets, which affect cellular migration, angiogenesis, and nutrient transfer—all essential processes in placental functioning. In addition, *miR-10b* was shown to be functional *in vitro* within the HTR-8 trophoblasts. The specific sequence binding and subsequent suppression of mRNA translation sets the stage for further studies evaluating the expression of known mRNA targets of *miR-10b*. *miR-149* has interesting potential target proteins that have been shown to be affected in human IUGR, and validated by our in-vitro work here. Taken together, these *in vitro* studies imply a functional importance to these miRNAs in placental nutrient insufficiency leading to IUGR.

Intrauterine growth restriction is a significant cause of perinatal morbidity and mortality with consequences extending into childhood and adulthood. This pregnancy complication remains poorly understood, with likely multiple regulatory inputs affecting fetal outcomes. It therefore becomes important to not only understand genetic regulation underlying placental insufficiency in IUGR, but also to discover methods for detecting at risk fetuses earlier in the pregnancy to allow for increased surveillance and intervention, if possible.

## Supporting information

S1 Fig Heatmap of top miRNA candidates down-regulated in SGA/IUGR placentas compared to control placentas. Red indicates up-regulation and green indicates down-regulation of *miRNA* expression. This heatmap represents good differentiation of SGA/IUGR from AGA (individual samples listed across the x-axis) based upon the 44 *miRNAs* identified as down-regulated in the SGA/IUGR group by microarray (depicted along the y-axis).
(PDF)

S2 Fig miR expression after 50% nutrient restriction in HTR8 trophoblast cells at 0, 1, 3, 6, and 24 hrs. (A) *miR-10b* expression, (B) *miR-363* expression, and (C) *mir-149* expression at baseline and after 1, 3, 6, and 24 hours of NR in HTR8 trophoblast cells. All data are expression as means ± SD. Asterisks denote significance, with p-value<0.05 by ANOVA for all time points.
(PDF)

S3 Fig Heatmap of top miRNA candidates up-regulated in SGA/IUGR placentas compared to AGA placentas in the expanded validation microarray. Red indicates up-regulation and green indicates down-regulation of miRNA expression. This heatmap represents good differentiation of SGA/IUGR from AGA (individual samples listed across the x-axis) based upon the 28 miRNAs identified as up-regulated in the SGA/IUGR group by validation microarray (depicted along the y-axis).
(PDF)

S1 Table qRT-PCR conditions. Primer and probe sequences, and annealing temperatures used for qRT-PCR experiments.
(PDF)

S2 Table Placental sample characteristics for AGA and IUGR placentas. Clinical characteristics of placental samples used, including a subset of IUGR-associated placentas with notable prenatal ultrasound findings. Numbers in parenthesis indicate percentiles for age (for weight, length and head circumference columns) M = male. F = female. NSVD = normal spontaneous vaginal delivery. C/S = cesarean section. HC = head circumference. FGD = fetal growth deceleration. UA = umbilical artery. MCA = middle cerebral artery. NR = data not recorded.
(PDF)

S3 Table miRNAs that were significantly up-regulated in small for gestational age (SGA)/IUGR placentas compared to appropriate for gestational age (AGA) placentas. Rank column indicates position in ranking by significance value. EB = Empirical Bayes’.
(PDF)

## References

[1] Longo S, Bollani L, Decembrino L, Di Comite A, Angelini M, Stronati M. Short-term and long-term sequelae in intrauterine growth retardation (IUGR). J Matern Fetal Neonatal Med. 2013; 26:222–225. 10.3109/14767058.2012.715006 23030765

[2] Devaskar SU, Chu A. Intrauterine growth restriction: hungry for an answer. Physiology (Bethesda). 2016; 31(2):131–146. 26889018 10.1152/physiol.00033.2015 PMC4895444

[3] Spinillo A, Capuzzo E, Egbe TO, Fazzi E, Colonna L, Nicola S. Pregnancies complicated by idiopathic intrauterine growth retardation. Severity of growth failure, neonatal morbidity and two-year infant neurodevelopmental outcome. J Reprod Med. 1999; 40(3):209–215. 7539849

[4] Pallotto EK, Kilbride HW. Perinatal outcome and later implications of intrauterine growth restriction. Clin Obstet Gynecol. 2006; 49(2):257–269. 16721105 10.1097/00003081-200606000-00008

[5] Leitner Y, Fattal-Valevski A, Geva R, Eshel R, Toledano-Alhadef H, Rotstein M, et al Neurodevelopmental outcome of children with intrauterine growth retardation: a longitudinal, 10-year prospective study. J Child Neurol. 2007; 22(5):580–587. 10.1177/0883073807302605 17690065

[6] Simić Klarić A, Kolundžić Z, Galić S, Mejaški Bošnjak V. Language development in preschool children born after asymmetrical intrauterine growth retardation. Eur J Paediatr Neurol. 2012; 16(2):132–137. 10.1016/j.ejpn.2011.06.003 21764612

[7] Geva R, Eshel R, Leitner Y, Valevski AF, Harel S. Neuropsychological outcome of children with intrauterine growth restriction: a 9-year prospective study. Pediatrics. 2006; 118(1):91–100. 10.1542/peds.2005-2343 16818553

[8] Chu A, De Beritto T. The perinatal origins of cardiovascular disease. Pediatr Ann. 2015; 44(11):e254–259. 10.3928/00904481-20151112-07 26587817

[9] Barker DJ, Bull AR, Osmond C, Simmonds SJ. Fetal and placental size and risk of hypertension in adult life. BMJ. 1990; 301(6746):259–62. 2390618 10.1136/bmj.301.6746.259 PMC1663477

[10] Nicholas LM, Morrison JL, Rattanatray L, Zhang S, Ozanne SE, McMillen IC. The early origins of obesity and insulin resistance: timing, programming and mechanisms. Int J Obes (Lond). 2016; 40(2):229–238. 26367335 10.1038/ijo.2015.178

[11] Dong Y, Zhang L, Zhang S, Bai Y, Chen H, Sun X, et al Phosphatase of regenerating liver 2 (PRL2) is essential for placental development by down-regulating PTEN (Phosphatase and Tensin Homologue deleted on chromosome 10) and activating Akt protein. J Biol Chem. 2012; 287(38):32172–9. 10.1074/jbc.M112.393462 22791713 PMC3442547

[12] Sandovici I, Hoelle K, Angiolini E, Constância M. Placental adaptations to the maternal-fetal environment: implications for fetal growth and developmental programming. Reprod Biomed Online. 2012; 25(1):68–89. 10.1016/j.rbmo.2012.03.017 22560117

[13] Ganguly A, Chen Y, Shin BC, Devaskar SU. Prenatal caloric restriction enhances DNA methylation and MeCP2 recruitment with reduced murine placental glucose transporter isoform 3 expression. J Nutr Biochem. 2014;25(2):259–66. 10.1016/j.jnutbio.2013.10.015 24445052 PMC4208916

[14] Janzen C, Lei MY, Cho J, Sullivan P, Shin BC, Devaskar SU. Placental glucose transporter 3 (GLUT3) is up-regulated in human pregnancies complicated by late-onset intrauterine growth restriction. Placenta. 2013;34(11):1072–8. 10.1016/j.placenta.2013.08.010 24011442 PMC3843645

[15] Barad O, Meiri E, Avniel A, Aharonov R, Barzilai A, Bentwich I, et al MicroRNA expression detected by oligonucleotide microarrays: system establishment and expression profiling in human tissues. Genome Res. 2004; 14(12):2486–94. 10.1101/gr.2845604 15574827 PMC534673

[16] Liang Y, Ridzon D, Wong L, Chen C. Characterization of microRNA expression profiles in normal human tissues. BMC Genomics. 2007; 8:166 10.1186/1471-2164-8-166 17565689 PMC1904203

[17] Morales-Prieto DM, Chaiwangyen W, Ospino-Prieto S, Schneider U, Hermann J, Gruhn B, Markert UR. MicroRNA expression profiles of trophoblastic cells. Placenta. 2012; 33(9):725–34. 10.1016/j.placenta.2012.05.009 22721760

[18] Ganguly A, Touma M, Thamotharan S, De Vivo DC, Devaskar SU. Maternal calorie restriction causing utero-placental insufficiency differentially affects mammalian placental glucose and leucine transport molecular mechanisms. Endocrinology. 2016; 157(10):4041–4054. 10.1210/en.2016-1259 27494059 PMC5045505

[19] Kim VN. MicroRNA biogenesis: coordinated cropping and dicing. Nat Rev Mol Cell Biol. 2005; 6(5):376–85. 10.1038/nrm1644 15852042

[20] Luo SS, Ishibashi O, Ishikawa G, Katayama A, Mishima T, Takizawa T, et al Human villous trophoblasts express and secrete placenta-specific microRNAs into maternal circulation via exosomes. Biol Reprod. 2009; 81(4):717–29. 10.1095/biolreprod.108.075481 19494253

[21] Mouillet JF, Chu T, Hubel CA, Nelson DM, Parks WT, Sadovsky Y. The levels of hypoxia-regulated microRNAs in plasma of pregnant women with fetal growth restriction. Placenta. 2010; 31(9):781–4. 10.1016/j.placenta.2010.07.001 20667590 PMC3204658

[22] Maccani MA, Padbury JF, Marsit CJ. miR-16 and miR-21 expression in the placenta is associated with fetal growth. PLoS One. 2011;6(6):e21210 10.1371/journal.pone.0021210 21698265 PMC3115987

[23] Wang W, Feng L, Zhang H, Hachy S, Satohisa S, Laurent LC, et al Preeclampsia up-regulates angiogenesis-associated microRNA (i.e. miR-17, -20a, and -20b) that target ephrin-B2 and EPHB4 in human placenta. J Clin Endocrinol Metab. 2012; 97(6):E1051–9. 10.1210/jc.2011-3131 22438230 PMC3387422

[24] Enquobahrie DA, Abetew DF, Sorensen TK, Willoughby D, Chidambaram K, Williams MA. Placental microRNA expression in pregnancies complicated by preeclampsia. Am J Obstet Gynecol. 2011; 204(2):e12–21. 10.1016/j.ajog.2010.09.004 PMC3040986 21093846

[25] Livak KJ, Schmittgen TD. Analysis of relative gene expression data using real-time quantitative PCR and the 2(-Delta Delta C(T)) method. Methods. 2001; 25(4):402–8. 10.1006/meth.2001.1262 11846609

[26] Ganguly A, McKnight RA, Raychaudhuri S, Shin BC, Ma Z, Moley K, Devaskar SU. Glucose transporter isoform-3 mutations cause early pregnancy loss and fetal growth restriction. Am J Physiol Endocrinol Metab. 2007,292(5):E1241–55. 10.1152/ajpendo.00344.2006 17213475

[27] Sankar R, Thamotharan S, Shin D, Moley KH, Devaskar SU. Insulin-responsive glucose transporters-GLUT8 and GLUT4 are expressed in the developing mammalian brain. Brain Res Mol Brain Res. 2002; 107(2):157–65. 12425944 10.1016/s0169-328x(02)00487-4

[28] Gierman LM, Stødle GS, Tangerâs LH, Austdal M, Olsen GD, Follestad T, et al Toll-like receptor profiling of seven trophoblast cell lines warrants caution for translation to primary trophoblasts. Placenta. 2015;36(11):1246–53. 10.1016/j.placenta.2015.09.004 26386649

[29] Chen Z, He P, Ding X, Huang Y, Gu H, Ni X. PPARϒ stimulates expression of L-type amino acid and taurine transporters in human placentas; the evidence of PPARϒ regulating fetal growth. Sci Rep. 2015; 5:12650 10.1038/srep12650 26227476 PMC4521151

[30] Dweep H, Gretz N. miRWalk2.0: a comprehensive atlas of microRNA-target interactions. Nat Methods. 2015; 12(8):687. 10.1038/nmeth.3485 26226356

[31] Smyth GK. Linear models and empirical bayes methods for assessing differential expression in microarray experiments. Stat Appl Genet Mol Biol. 2004; 3(3). 10.2202/1544-6115.1027 16646809

[32] Guo L, Tsai SQ, Hardison NE, James AH, Motsinger-Reif AA, Thames B, et al Differentially expressed microRNAs and affected biological pathways revealed by modulated modularity clustering (MMC) analysis of human preeclamptic and IUGR placentas. Placenta. 2013;34(7):599–605. 10.1016/j.placenta.2013.04.007 23639576 PMC3677766

[33] Chim SS, Shing TK, Hung EC, Leung TY, Lau TK, Chiu RW, Lo YM. Detection and characterization of placental microRNAs in maternal plasma. Clin Chem. 2008;54(3):482–90. 10.1373/clinchem.2007.097972 18218722

[34] Benjamini Y, Hochberg Y. “Controlling the false discovery rate: a practical and powerful approach to multiple testing.” J Royal Stat Soc Series B. 1995;57:289–300.

[35] Shi XF, Wang H, Xiao FJ, Yin Y, Xu QQ, Ge RL, Wang LS. “MiRNA-486 regulates angiogenic activity and survival of mesenchymal stem cells under hypoxia through modulating Akt signal.” Biochem Biophys Res Commun. 2016;470(3):670–7. 10.1016/j.bbrc.2016.01.084 26801559

[36] Liu L, Zang J, Chen X, Yang G, Zhu Y, Wu Y, Li T. “Role of miR-124 and miR-141 in the regulation of vascular reactivity and the relationship to RhoA and Rac1 after hemorrhage and hypoxia.” Am J Physiol Heart Circ Physiol. 2016;310(2):H206–16. 10.1152/ajpheart.00651.2014 26453334

[37] Sun X, Zuo H, Liu C, Yang Y. “Overexpression of miR-200a protects cardiomyocytes against hypoxia-induced apoptosis by modulating the kelch-like ECH-associated protein 1-nuclear factor erythroid 2-related factor 2 signaling axis.” Int J Mol Med. 2016;38(4):1303–11. 10.3892/ijmm.2016.2719 27573160

[38] Zhang H, Guo X, Feng X, Wang T, Hu Z, Que X, Tian Q, Zhu T, Guo G, Huang W, Li X. “MiRNA-543 promotes osteosarcoma cell proliferation and glycolysis by partially suppressing PRMT9 and stabilizing HIF-1a protein.” Oncotarget. 2016 [epub ahead of print]. 10.18632/oncotarget.13672 PMC5356804 27911265

[39] Zhu HJ, Han ZY, He SF, Jin SY, Xu SJ, Fang XD, Zang Y. “Specific MicroRNAs comparisons in hypoxia and morphine preconditioning again hypoxia-reoxygenation injury with and without heart failure.” Life Sci. 2016;S0024-3205(16)30680-4. 10.1016/j.lfs.2016.11.028 27919821

[40] Zhang W, Shang T, Huang C, Yu T, Liu C, Qiao T, Huang D, Liu Z, Liu C. “Plasma microRNAs serve as potential biomarkers for abdominal aortic aneurysm.” Clin Biochem. 2015;48(150):988–92. 25916817 10.1016/j.clinbiochem.2015.04.016

[41] Yang B, Jing C, Wang J, Guo X, Chen Y, Xu R, Peng L, Liu J, Li L. “Identification of microRNAs associated with lymphangiogenesis in human gastric cancer.” Clin Transl Oncol. 2014;16(4):374–9 10.1007/s12094-013-1081-6 23881463

[42] Qin B, Shu Y, Xiao L, Lu T, Lin Y, Yang H, Lu Z. “MicroRNA-150 targets ELK1 and modulates the apoptosis induced by ox-LDL in endothelial cells.” Mol Cell Biochem. 2017;[Epub ahead of print]. 10.1007/s11010-016-2935-3 28110404

[43] Ramalinga M, Roy A, Srivastava A, Bhattarai A, Harish V, Suy S, Collins S, Kumar D. “MicroRNA-212 negatively regulates starvation induced autophagy in prostate cancer cells by inhibiting SIRT1 and is a modulator of angiogenesis and cellular senescence.” Oncotarget. 2015;6(33):34446–57. 10.18632/oncotarget.5920 26439987 PMC4741465

[44] Joseph G, Soler A, Hutcheson R, Hunter I, Bradford C, Hutcheson B, Gotlinger KH, Jiang H, Falck JR, Proctor S, Laniado Schwartzman M, Rocic P. “Elevated 20-HETE impairs coronary collateral growth in metabolic syndrome via endothelial dysfunction.” Am J Physiol Heart Circ Physiol. 2016;[epub ahead of print]. 10.1152/ajpheart.00561.2016 PMC5402017 28011587

[45] De Jong VM, Zaldumbide A, van der Slik AR, Persengiev SP, Roep BO, Koeleman BP. “Post-transcriptional control of candidate risk genes for type 1 diabetes by rare genetic variants.” Genes Immun. 2013;14(1):58–61. 10.1038/gene.2012.38 22932817

[46] Rotllan N, Fernández-Hernando C. “MicroRNA regulation of cholesterol metabolism.” Cholesterol. 2012;847849 10.1155/2012/847849 22919472 PMC3420088

[47] Yan S, Wang T, Huang S, Di Y, Huang Y, Liu X, Luo Z, Han W, An B. “Differential expression of microRNAs in plasma of patients with prediabetes and newly diagnosed type 2 diabetes.” Acta Diabetol. 2016;53(5):693–702. 10.1007/s00592-016-0837-1 27039347

[48] Delić D, Eisele C, Schmid R, Baum P, Wiech F, Gerl M, Zimdahl H, Pullen SS, Urquhart R. “Urinary exosomal miRNA signature in type II diabetic nephropathy patients.” PLoS One. 2016;11(3):e0150154 10.1371/journal.pone.0150154 26930277 PMC4773074

[49] Labialle S, Marty V, Bortolin-Cavaillé ML, Hoareau-Osman M, Pradère JP, Valet P, Martin PG, Cavaillé J. EMBO J. “The miR-379/miR-410 cluster at the imprinted Dlk1-Dio3 domain controls neonatal metabolic adaptation.” 2014;33(19):2216–30. 10.15252/embj.201387038 25124681 PMC4282508

[50] Sun Y, Peng R, Peng H, Liu H, Wen L, Wu T, Yi H, Li A, Zhang Z. “miR-451 suppresses the NF-kappaB-mediated proinflammatory molecules expression through inhibiting LMP7 in diabetic nephropathy.” Mol Cell Endocrinol. 2016;433:75–86. 10.1016/j.mce.2016.06.004 27264074

[51] Xu Z, Dong D, Chen X, Huang H, Wen S. “MicroRNA-381 negatively regulated TLR4 signaling in A549 cells in response to LPS stimulation.” Biomed Res Int. 2015;849475 10.1155/2015/849475 26688820 PMC4672107

[52] Li Q, Long A, Jiang L, Cai L, Xie LI, Gu J, et al Quantification of preeclampsia-related microRNAs in maternal serum. Biomed Rep. 2015;3(6):792–796. 10.3892/br.2015.524 26623017 PMC4660598

[53] Zhang C, Li Q, Ren N, Li C, Wang X, Xie M, Gao Z, Pan Z, Zaheo C, Ren C, Yang W. “Placental miR-106a~363 closter is dysregulated in preeclamptic placenta.” Placenta. 2015;36(2):250–2. 10.1016/j.placenta.2014.11.020 25499681

[54] Xu P, Zhao Y, Liu M, Wang Y, Wang H, Li YX, Zhu X, Yao Y, Wang H, Qiao J, Ji L, Wang YL. “Variations of microRNAs in human placentas and plasma from preeclamptic pregnancy.” Hypertension. 2014;63(6):1276–84. 10.1161/HYPERTENSIONAHA.113.02647 24664294

[55] Hromadnikova I, Kotlabova K, Ondrackova M, Pirkova P, Kestlereova A, Novotna V, Hympanova L, Krofta L. “Expression profile of C19MC microRNAs in placental tissue in pregnancy-related complications. DNA Cell Biol. 2015;34(6):437–57. 10.1089/dna.2014.2687 25825993 PMC4486149

[56] Sadeghi M, Ranjbar B, Ganjalikhany MR, M Khan F, Schmitz U, Wolkenhauer O, Gupta SK. “MicroRNA and transcription factor gene regulatory network analysis reveals key regulatory elements associated with prostate cancer progression.” PLoS One. 2016;11(12):e0168760 10.1371/journal.pone.0168760 28005952 PMC5179129

[57] Stojadinovic O, Ramirez H, Pastar I, Gordon KA, Stone R, Choudhary S, Badiavas E, Nouri K, Tomic-Canic M. MiR-21 and miR-205 are induced in invasive cutaneous squamous cell carcinomas. Arch Dermatol Res. 2016;[epub ahead of print]. 10.1007/s00403-016-1705-0 28013372

[58] Wang Z, Li Q. Chamba Y, Zahng B, Shang P, Zhang H, Wu C. “Identification of genes related to growth and lipid deposition from transcriptom profiles of pig muscle tissue.” PLoS One. 2015;10(10):e0141138 10.1371/journal.pone.0141138 26505482 PMC4624711

[59] Wang D, Na Q, Song WW, Song GY. “Altered expression of miR-518b and miR-519a in the placenta is associated with low fetal birth weight.” Am J Perinatol. 2014;31(9):729–34. 10.1055/s-0033-1361832 24683074

[60] Yang S, Li H, Ge Q, Guo L, Chen F. “Dergulated microRNA species in the plasma and placenta of patients with preeclampsia.” Mol Med Rep. 2015;12(10):527–34). 25738738 10.3892/mmr.2015.3414

[61] Hromadnikova I, Kotlabova K, Hympanova L, Krofta L. “Cardiovascular and cerebrovascular disease associated microRNAs are dysregulated in placental tissues affected with gestational hypertension, preeclampsia and intrauterine growth restriction.” PLoS One. 2015;10(9):e0138383 10.1371/journal.pone.0138383 26394310 PMC4579085

[62] Ackerman WE 4th, Buhimschi IA, Eidem HR, Rinker DC, Rokas A, Rood K, et al Comprehensive RNA profiling of villous trophoblast and decidua basalis in pregnancies complicated by preterm birth following intra-amniotic infection. Placenta. 2016; 44:23–33. 10.1016/j.placenta.2016.05.010 27452435 PMC11583243

[63] Vashukova ES, Glotov AS, Fedotox PV, Efimova OA, Pakin VS, Mozgovaya EV, et al Placental microRNA expression in pregnancies complicated by superimposed pre-eclampsia on chronic hypertension. Mol Med Rep. 2016;14(1):22–32. 10.3892/mmr.2016.5268 27176897 PMC4918533

[64] Santa LM, Teshima LY, Forero JV, Giraldo AO. AngiomiRs: potential biomarkers of pregnancy’s vascular pathologies. J Pregnancy. 2015; 320386 10.1155/2015/320386 26550492 PMC4621355

[65] Liu ZY, Zhao J, Zhang PY, Zhang Y, Sun SY, Yu SY, Xi QS. MicroRNA 10b targets E-cadherin and modulates breast cancer metastasis. Med Sci Monit. 2012; 18(8):299–308. 10.12659/MSM.883262 PMC3560697 22847191

[66] Li YZ, Wen L, Wei X, Wang QR, Xu LW, Zhang HM, Liu WC. Inhibition of miR-7 promotes angiogenesis in human umbilical vein endothelial cells by upregulating VEGF via KLF4. Oncol Rep. 2016;36(3):1569–75. 10.3892/or.2016.4912 27431648

[67] Wang Y, Yang C, Gu Q, Sims M, Gu W, Pfeffer LM, Yue J. KLF4 promotes angiogenesis by activating VEGF signaling in human retinal microvascular endothelial cells. PLoS One. 2015; 10(6):e0130341 10.1371/journal.pone.0130341 26075898 PMC4467843

[68] Hale AT, Tian H, Anih E, Recio FO 3rd, Shata MA, Johnson T, et al Endothelial kruppel-like factor 4 regulated angiogenesis and the Notch signaling pathway. J Biol Chem. 2014;289(17):12016–28. 10.1074/jbc.M113.530956 24599951 PMC4002108

[69] Zhu XM, Han T, Sargent IL, Yin GW, Yao YQ. Differential expression profile of microRNAs in human placentas from preeclamptic pregnancies versus normal pregnancies. Am J Obstet Gynecol. 2009; 200:661.e1–7. 19285651 10.1016/j.ajog.2008.12.045

[70] Ganguly A, Collis L, Devaskar SU. Placental glucose and amino acid transport in calorie-restricted wild-type and Glut3 null heterozygous mice. Endocrinology. 2012;153(8):3995–4007. 10.1210/en.2011-1973 22700768 PMC3404359

[71] Rosario FJ, Jansson N, Kanai Y, Prasad PD, Powerll TL, Jansson T. Maternal protein restriction in the rat inhibits placental insulin, mTOR, and STAT3 signaling and down-regulates placental amino acid transporters. Endocrinology. 2001;152(30):1119–29. 10.1210/en.2010-1153 PMC3858644 21285325

[72] Kavitha JV, Rosario FJ, Nijland MJ, McDonald TJ, Wu G, Kanai Y, et al Down-regulation of placental mTOR, insulin/IGF-1 signaling, and nutrient transporters in response to maternal nutrient restriction in the baboon. FASEB J. 2014;28(3):1294–305. 10.1096/fj.13-242271 24334703 PMC3929672

[73] Chen YY, Rosario FJ, Shehab MA, Powerll TL, Gupta MB, Jansson T. Increased ubiquitination and reduced plasma membrane trafficking of placental amino acid transporter SNAT-2 in human IUGR. Clin Sci (Lond). 2015;129(12):1131–41. 26374858 10.1042/CS20150511 PMC4614027

[74] Roos S, Lagerlof O, Wennergren M, Powell TL, Jansson T. Regulation of amino acid transporters by glucose and growth factors in cultured primary human trophoblast cells is mediated by mTOR signaling. Am J Physiol Cell Physiol. 2009;297(3):C723–31. 10.1152/ajpcell.00191.2009 19587219

[75] Roos S, Jansson N, Palmberg I, Säljö K, Powerll TL, Jansson T. Mammalian target of rapamycin in the human placenta regulated leucine transport and is down-regulated in restricted fetal growth. J Physiol. 2007;582(pt 1):449–59. 17463046 10.1113/jphysiol.2007.129676 PMC2075295

[76] Day PE, Ntani G, Crozier SR, Mahon PA, Inskip HM, Cooper C, et al Maternal factors are associated with the expression of placental genes involved in amino acid metabolism and transport. PLoS One. 2015;10(12):e0143653 10.1371/journal.pone.0143653 26657885 PMC4682815

[77] Lager S, Powell TL. Regulation of nutrient transport across the placenta. 2012. J Pregnancy. 2012;2012:179827 10.1155/2012/179827 23304511 PMC3523549

