# Supplementary figures and images for: Differential microRNA expression in human placentas of term intra-uterine growth restriction that regulates target genes mediating angiogenesis and amino acid transport
Source: PLoS One. 2017 May 2;12(5):e0176493. doi: 10.1371/journal.pone.0176493 (PMC5413012; doi:10.1371/journal.pone.0176493)

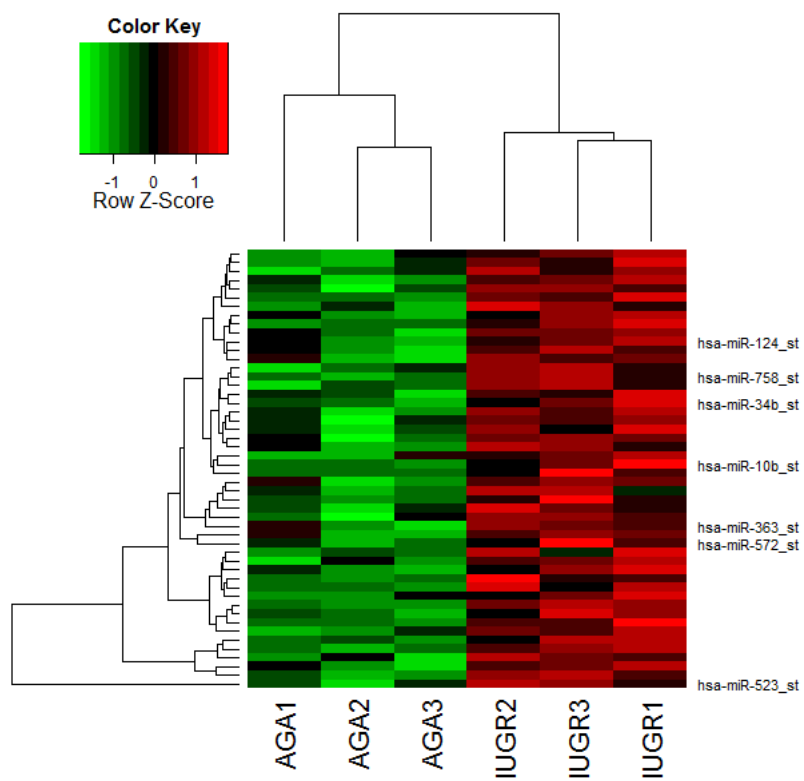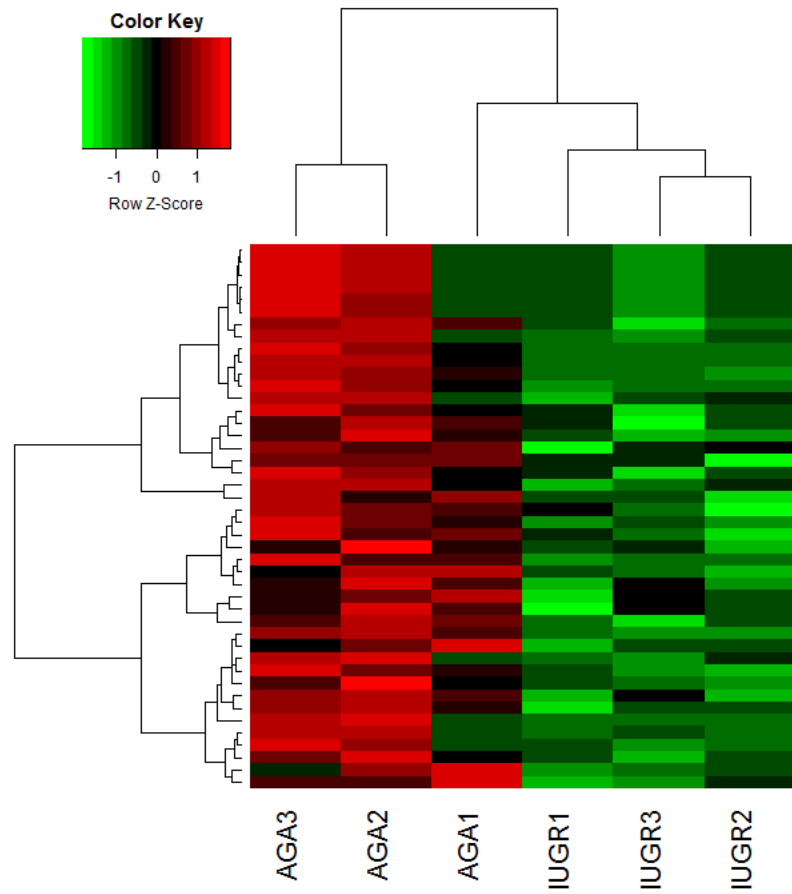

Supplement: S1 Fig — Red indicates up-regulation and green indicates down-regulation of miRNA expression. This heatmap represents good differentiation of SGA/IUGR from AGA (individual samples listed across the x-axis) based upon the 44 miRNAs identified as down-regulated in the SGA/IUGR group by microarray (depicted along the y-axis). (PDF) [file pone.0176493.s001.pdf]

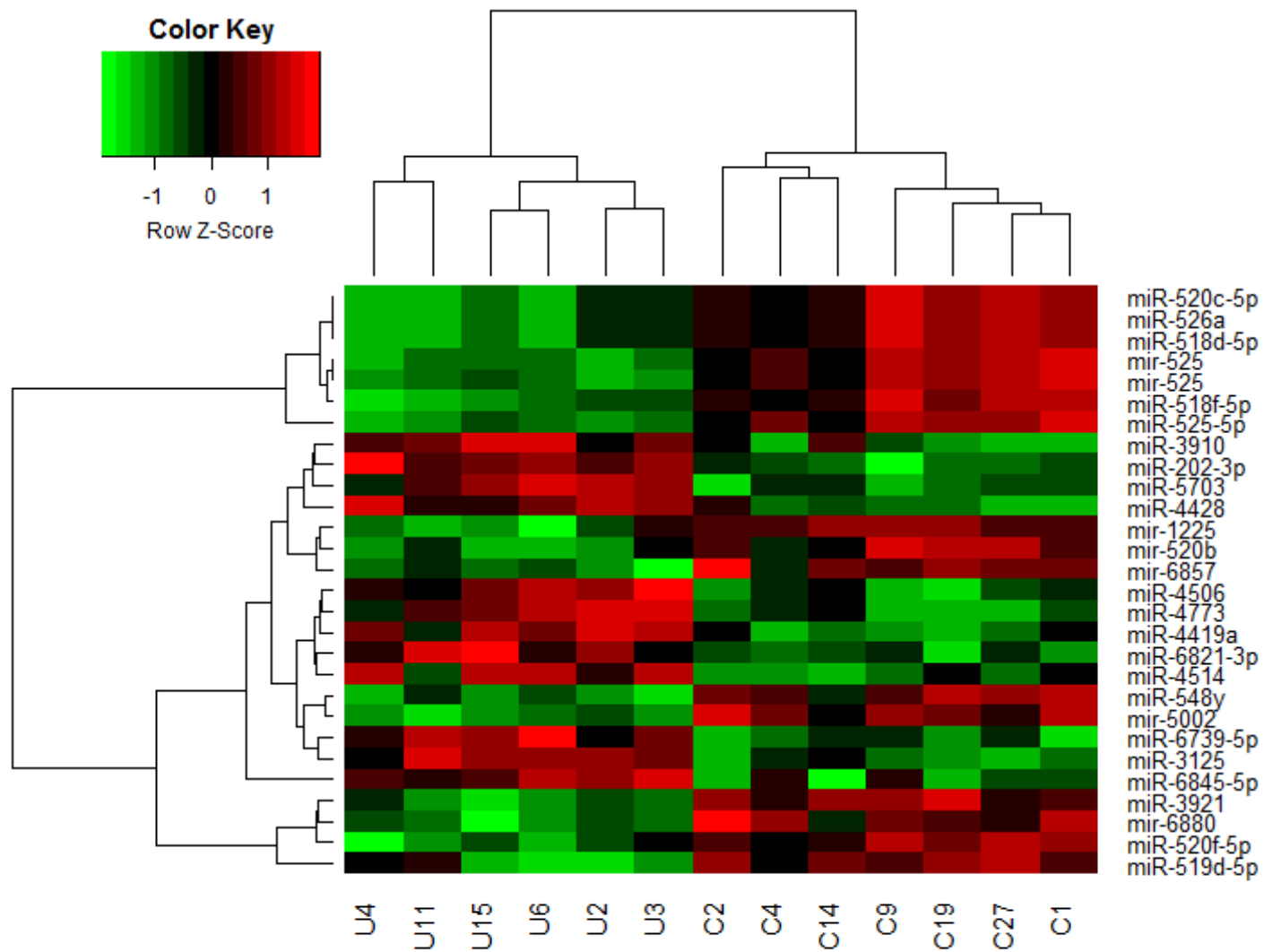

Supplement: S3 Fig — Red indicates up-regulation and green indicates down-regulation of miRNA expression. This heatmap represents good differentiation of SGA/IUGR from AGA (individual samples listed across the x-axis) based upon the 28 miRNAs identified as up-regulated in the SGA/IUGR group by validation microarray (depicted along the y-axis). (PDF) [file pone.0176493.s003.pdf]
