# Supplementary material for: Differential microRNA expression in human placentas of term intra-uterine growth restriction that regulates target genes mediating angiogenesis and amino acid transport
Source: PLoS One. 2017 May 2;12(5):e0176493. doi: 10.1371/journal.pone.0176493 (PMC5413012; doi:10.1371/journal.pone.0176493)

S2 Fig

2A

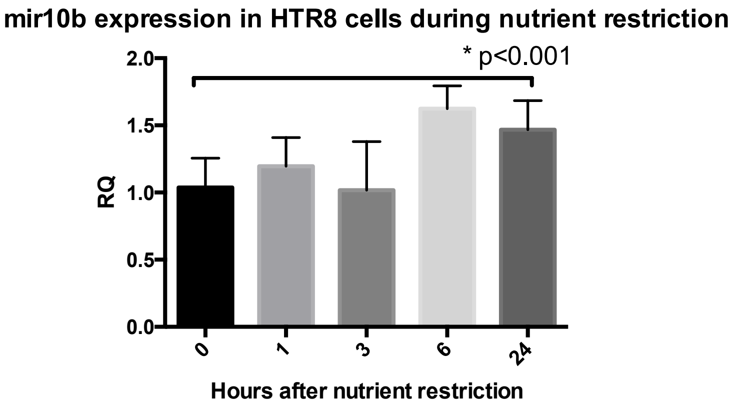

2B

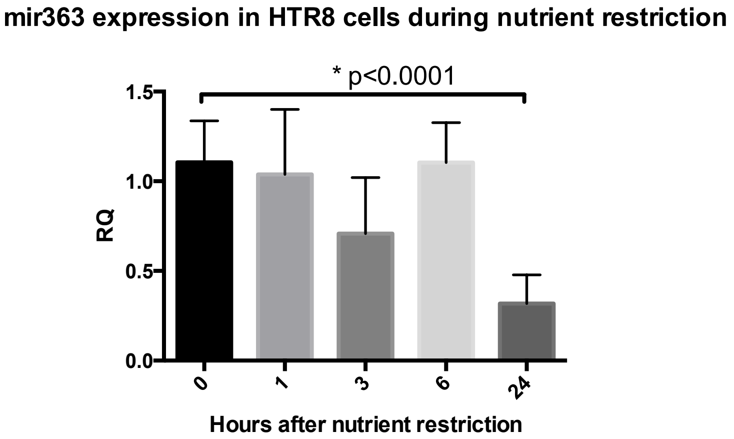

2C

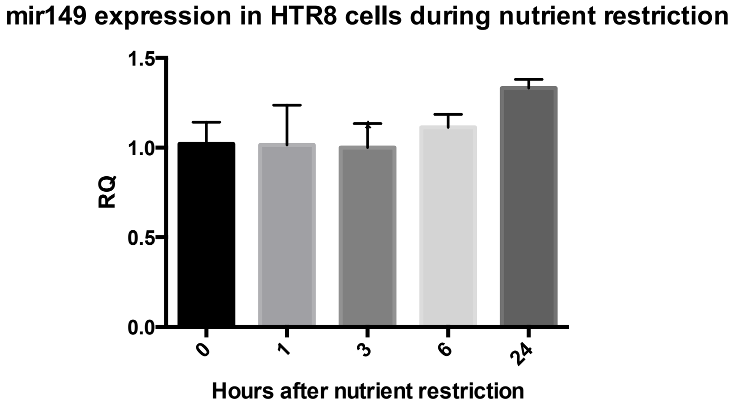

Supplement: S2 Fig — (A) miR-10b expression, (B) miR-363 expression, and (C) mir-149 expression at baseline and after 1, 3, 6, and 24 hours of NR in HTR8 trophoblast cells. All data are expression as means ± SD. Asterisks denote significance, with p-value<0.05 by ANOVA for all time points. (PDF) [file pone.0176493.s002.pdf]
