# Supplementary material for: Differential microRNA expression in human placentas of term intra-uterine growth restriction that regulates target genes mediating angiogenesis and amino acid transport
Source: PLoS One. 2017 May 2;12(5):e0176493. doi: 10.1371/journal.pone.0176493 (PMC5413012; doi:10.1371/journal.pone.0176493)
Supplement: S1 Table — Primer and probe sequences, and annealing temperatures used for qRT-PCR experiments. (PDF) [file pone.0176493.s004.pdf]

# S1 Table

| Gene | Annealing temperature | Forward primer | Reverse primer | Probe |
|---|---|---|---|---|
| **e-cadherin** | 58°C | 5'-TTCCAGGAACCTCTGTGATG | 5'-TCTTGGCTGAGGATGGTGTA | 5'-CATCGTCCGCGTCTGTGGCT |
| **HOXD10** | 56°C | 5'-GGAAAGCAAAGAGGAAATCAA | 5'-CAGCGTTTGGTGCTTAGTGT | 5'-TGCCACTCTTTGCAGTGAGCCA |
| **SNAT1** | 58°C | 5'-GCGGCTGGCTGTCATTG | 5'-AAAGATGAACGAACCGTGAAAAATA | 5'-TGCTGTGATCCTCACAGTGCCGGT |
| **SNAT2** | 60°C | 5'- GACAGCAGCAGCTACAGTTCCA | 5'-ATGGCTTTTCAGAGCAGCTTG | 5'-CAGCGACTTCAACTACTCCTACCCCACCA |
| **IR** | 60°C | 5'-CCTCCTAGAAGGCGAGAAGA | 5'-AGATTGTTGCCTCCTCGAAT | 5'-CCCAGGAGCTCCGAGGATGC |
| **LAT2** | 58°C | 5'-CGGAGTAGCCCTGAAGAAAG | 5'-CGATGATGTTCCCTACGATG | 5'-CCACAGGCACTGACCAATCCG |
| **EGFR** | 58°C | 5'-GAACTGCCAGAAACTGACCA | 5'-ACTGGTTGTGGCAGCAGTC | 5'-CTGTGCCCAGCAGTGCTCCG |
