# Supplementary material for: Differential microRNA expression in human placentas of term intra-uterine growth restriction that regulates target genes mediating angiogenesis and amino acid transport
Source: PLoS One. 2017 May 2;12(5):e0176493. doi: 10.1371/journal.pone.0176493 (PMC5413012; doi:10.1371/journal.pone.0176493)
Supplement: S2 Table — Clinical characteristics of placental samples used, including a subset of IUGR-associated placentas with notable prenatal ultrasound findings. Numbers in parenthesis indicate percentiles for age (for weight, length and head circumference columns) M = male. F = female. NSVD = normal spontaneous vaginal delivery. C/S = cesarean section. HC = head circumference. FGD = fetal growth deceleration. UA = umbilical artery. MCA = middle cerebral artery. NR = data not recorded. (PDF) [file pone.0176493.s005.pdf]

S2 Table

	Sample	Gestational age at birth (weeks)	Sex of infant	Delivery mode	Birthweight (g)	Length (cm)	Head circumference (cm)	Notable Prenatal ultrasound findings
**AGA**	C1	41+5	M	NSVD	3795 (40%)	55.9 (95%)	34.3 (10%)	
	C2	38+0	M	C/S	2778 (20%)	49.5 (60%)	35.6 (70%)	
	C3	39+1	M	NSVD	2760 (10%)	45.72 (3%)	33.9 (10%)	
	C4	39+5	M	NSVD	3260 (30%)	50.8 (50%)	32 (5%)	
	C5	38+1	M	C/S	3231 (50%)	49.5 (50%)	33.9 (50%)	
	C7	39+4	F	C/S	3940 (80%)	52.1 (75%)	35.6 (50%)	
	C8	39+0	F	C/S	3544 (73%)	49.5 (50%)	34.92 (50%)	
	U8	39+3	F	NSVD	2985 (20%)	49.5 (40%)	31.4 (<3%)	
	U9	38+1	F	NSVD	2693 (15%)	48.3 (40%)	33 (20%)	
	C9	39+4	M	C/S	3713 (75%)	48.3 (20%)	36.8 (80%)	
	C10	38+0	M	C/S	3912 (86%)	53.34 (90%)	34.29 (50%)	
	C11	39+3	F	C/S	3600 (66%)	50.8 (60%)	35.56 (50%)	
	C12	39+0	F	C/S	3110 (23%)	46 (5%)	34 (10%)	
	C14	39+0	F	C/S	3460 (50%)	51 (60%)	35 (50%)	
	C15	39+5	F	C/S	3203 (20%)	52 (75%)	35.6 (50%)	
	C16	39+6	M	C/S	4054 (75%)	52.1 (75%)	34.3 (25%)	
	C17	39+2	M	C/S	4139 (90%)	52.1 (75%)	37.5 (90%)	
	C19	39	M	C/S	3600 (70%)	50.8 (50%)	38.1 (97%)	
	C24	37+5	F	C/S	3195 (66%)	NR	NR	
	C25	39+2	F	C/S	3275 (40%)	48.3 (25%)	35.6 (50%)	
	C27	37+0	M	C/S	2948 (50%)	NR	NR	
**IUGR**								
	U2	37+6	F	NSVD	2475 (8%)	48.3 (40%)	33 (10%)	FGD at 37 weeks
	U3	37+5	M	NSVD	2268 (3%)	46 (40%)	31 (5%)	
	C3	39+1	M	NSVD	2760 (9%)	45.7 (3%)	33.9 (25%)	
	U4	38+4	M	C/S	2551 (5%)	49.5 (50%)	33 (20%)	FGD at 37 weeks
	U5	38	F	C/S	2190 (<3%)	47 (15%)	30.5 (3%)	FGD at 34 weeks
	U6	40+2	M	C/S	2580 (<3%)	46.5 (<3%)	30.9 (<3%)	
	U10	39+5	F	NSVD	2863 (9%)	48.3 (15%)	30.5 (<3%)	
	U11	39+0	M	NSVD	2608 (4%)	44.5 (10%)	NR	
	U14	38	M	NSVD	2460 (6%)	43.2 (<3%)	30.5 (3%)	Abnormal UA and MCA Doppler, FGD at 37 weeks
	U15	38+6	M	NSVD	2645 (7%)	49 (25%)	32.5 (8%)	Abnormal MCA Doppler with brain sparing effect, FGD at 38 weeks
