# Supplementary material for: Differential microRNA expression in human placentas of term intra-uterine growth restriction that regulates target genes mediating angiogenesis and amino acid transport
Source: PLoS One. 2017 May 2;12(5):e0176493. doi: 10.1371/journal.pone.0176493 (PMC5413012; doi:10.1371/journal.pone.0176493)
Supplement: S3 Table — Rank column indicates position in ranking by significance value. EB = Empirical Bayes’. (PDF) [file pone.0176493.s006.pdf]

| Probe Set ID | Rank | Average expression level (AGA) | Average expression level (SGA/IUGR) | EB p-value |
|---|---|---|---|---|
| hsa-miR-1914-star_st | 2 | 6.0252 | 6.7953 | 0.00105 |
| hp_hsa-mir-523_st | 3 | 8.246 | 8.9238 | 0.00146 |
| hsa-miR-758_st | 4 | 5.4021 | 6.258 | 0.00189 |
| hsa-miR-4731-3p_st | 10 | 5.4899 | 6.2625 | 0.00329 |
| hsa-miR-889_st | 11 | 5.2232 | 5.961 | 0.00337 |
| hp_hsa-mir-1289-2_st | 16 | 5.4989 | 6.0573 | 0.00464 |
| hsa-miR-4798-3p_st | 17 | 5.9347 | 6.5182 | 0.00469 |
| hsa-miR-4486_st | 18 | 7.393 | 8.1732 | 0.00484 |
| hp_hsa-mir-486_x_st | 23 | 4.1467 | 4.702 | 0.00518 |
| hsa-miR-4677-3p_st | 27 | 5.8806 | 6.5631 | 0.006 |
| hp_hsa-mir-934_st | 28 | 6.4038 | 7.0603 | 0.00609 |
| hsa-miR-34b_st | 30 | 5.4445 | 6.2112 | 0.00658 |
| hsa-miR-10b_st | 32 | 5.4132 | 6.5779 | 0.00713 |
| hsa-miR-4741_st | 35 | 8.4075 | 9.3627 | 0.0076 |
| hsa-miR-4804-5p_st | 36 | 5.0422 | 5.6154 | 0.00794 |
| hsa-miR-2681_st | 38 | 5.3561 | 6.2403 | 0.00861 |
| hsa-miR-4319_st | 39 | 6.1967 | 7.2917 | 0.00882 |
| hsa-miR-1281_st | 42 | 5.3483 | 7.8855 | 0.0093 |
| hsa-miR-363_st | 43 | 6.9308 | 8.0587 | 0.00937 |
| hsa-miR-523_st | 46 | 10.3335 | 10.9996 | 0.00991 |
| hsa-miR-4418_st | 49 | 5.0329 | 5.4715 | 0.01038 |
| hsa-miR-411-star_st | 52 | 6.7527 | 7.5708 | 0.01108 |
| hsa-miR-3188_st | 54 | 6.6731 | 7.3644 | 0.01145 |
| hsa-miR-4743_st | 55 | 7.9451 | 8.4009 | 0.01147 |
| hsa-miR-4653-5p_st | 56 | 5.0058 | 5.7423 | 0.01201 |
| hsa-miR-4718_st | 57 | 5.4397 | 6.1482 | 0.01206 |
| hp_hsa-mir-3917_st | 59 | 6.2192 | 6.8539 | 0.0122 |
| hsa-miR-124_st | 60 | 6.159 | 6.992 | 0.01231 |
| hsa-miR-3619-5p_st | 65 | 6.4572 | 6.9669 | 0.01351 |
| hsa-miR-1911-star_st | 70 | 6.025 | 6.5778 | 0.01515 |
| hsa-miR-1180_st | 71 | 6.1464 | 7.026 | 0.01525 |
| hsa-miR-371b-5p_st | 76 | 5.6972 | 6.7046 | 0.01617 |
| hp_hsa-mir-524_x_st | 83 | 5.9072 | 6.2789 | 0.0177 |
| hsa-miR-4260_st | 84 | 5.5697 | 6.429 | 0.01771 |
| hsa-miR-588_st | 85 | 4.8511 | 5.3959 | 0.01772 |
| hp_hsa-mir-1299_st | 88 | 5.6525 | 6.182 | 0.01859 |
| hsa-miR-572_st | 89 | 5.6308 | 7.2146 | 0.01864 |
| hsa-miR-4737_st | 92 | 5.1888 | 5.9222 | 0.01931 |
| hp_hsa-mir-371b_st | 95 | 5.5779 | 5.9572 | 0.01961 |
| hsa-miR-3939_st | 98 | 6.5574 | 7.0978 | 0.02011 |
| hsa-miR-4252_st | 104 | 5.6977 | 6.5083 | 0.02083 |

| | | | | |
|---|---|---|---|---|
| hsa-miR-4725-3p_st | 108 | 6.734 | 7.2378 | 0.02094 |
| hsa-miR-200a_st | 109 | 5.2114 | 5.8923 | 0.02097 |
| hp_hsa-let-7d_st | 111 | 4.9574 | 5.6134 | 0.0213 |
| hsa-miR-4432_st | 112 | 4.6443 | 5.1496 | 0.02153 |
| hsa-miR-767-5p_st | 113 | 6.177 | 7.0255 | 0.02159 |
| hsa-miR-379-star_st | 114 | 4.768 | 5.3577 | 0.02218 |
| hsa-miR-1972_st | 115 | 5.5914 | 6.4774 | 0.02288 |
| hp_hsa-mir-543_st | 118 | 4.1415 | 4.4599 | 0.02382 |
| hsa-miR-4277_st | 119 | 5.6249 | 6.8739 | 0.02387 |
| hsa-miR-1915_st | 120 | 11.2014 | 11.9745 | 0.02398 |
| hsa-miR-4655-5p_st | 121 | 8.3018 | 8.7073 | 0.02417 |
| hsa-miR-451_st | 123 | 10.2624 | 11.1254 | 0.0246 |
| hp_hsa-mir-324_st | 124 | 5.4948 | 5.8733 | 0.02504 |
| hsa-miR-4784_st | 126 | 6.4237 | 6.7028 | 0.02521 |
| hp_hsa-mir-3960_st | 132 | 5.5016 | 6.084 | 0.02658 |
| hsa-miR-4699-5p_st | 134 | 4.6633 | 5.2382 | 0.02787 |
| hsa-miR-2861_st | 136 | 11.3633 | 11.9605 | 0.02809 |
| hp_hsa-mir-501_x_st | 139 | 5.3 | 5.7345 | 0.02844 |
| hsa-miR-4422_st | 140 | 5.7984 | 6.3041 | 0.03012 |
| hp_hsa-mir-381_st | 141 | 5.327 | 5.5975 | 0.0302 |
| hsa-miR-4520b-3p_st | 143 | 6.4887 | 7.0288 | 0.03111 |
| hsa-miR-4438_st | 147 | 5.5851 | 6.3387 | 0.03159 |
| hsa-miR-4466_st | 152 | 11.6365 | 12.1184 | 0.03274 |
| hsa-miR-4791_st | 154 | 4.3303 | 4.6789 | 0.03322 |
| hp_hsa-mir-548m_st | 157 | 5.1124 | 5.5384 | 0.03384 |
| hp_hsa-mir-523_x_st | 160 | 4.2356 | 4.482 | 0.03493 |
| hsa-miR-378-star_st | 161 | 5.6728 | 6.5013 | 0.03504 |
| hsa-miR-142-5p_st | 164 | 5.5332 | 6.0961 | 0.03576 |
| hsa-miR-378c_st | 165 | 9.1627 | 9.6994 | 0.03576 |
| hsa-miR-4299_st | 171 | 7.3205 | 7.9267 | 0.03653 |
| hsa-miR-3663-5p_st | 177 | 5.4984 | 6.0574 | 0.03713 |
| hp_hsa-mir-4430_st | 178 | 6.6151 | 6.8847 | 0.038 |
| hsa-miR-543_st | 181 | 9.1972 | 9.6655 | 0.03805 |
| hp_hsa-mir-3656_st | 182 | 7.1871 | 7.849 | 0.0387 |
| hsa-miR-2115-star_st | 183 | 5.181 | 5.8415 | 0.03921 |
| hsa-miR-638_st | 184 | 11.8239 | 12.5363 | 0.03921 |
| hsa-miR-33a-star_st | 185 | 5.3591 | 5.8742 | 0.03972 |
| hsa-miR-3690_st | 186 | 6.5683 | 7.0343 | 0.03991 |
| hsa-miR-150-star_st | 187 | 6.0838 | 6.8666 | 0.04006 |
| hsa-miR-422a_st | 189 | 7.7414 | 8.4353 | 0.04037 |
| hsa-miR-664-star_st | 192 | 7.1261 | 7.497 | 0.04079 |
| hsa-miR-212_st | 193 | 6.3762 | 6.7622 | 0.04084 |
| hsa-miR-3936_st | 194 | 5.6098 | 5.9897 | 0.04104 |
| hsa-miR-3975_st | 196 | 5.0116 | 5.4224 | 0.04167 |

| | | | | |
|---|---|---|---|---|
| hsa-miR-378f_st | 198 | 7.9499 | 8.91 | 0.04183 |
| hp_hsa-mir-561_x_st | 202 | 4.609 | 5.0953 | 0.04266 |
| hsa-miR-181d_st | 206 | 6.939 | 7.3689 | 0.0436 |
| hsa-miR-145_st | 211 | 14.2987 | 14.652 | 0.04445 |
| hsa-miR-4293_st | 212 | 6.1296 | 6.9609 | 0.04461 |
| hp_hsa-mir-138-1_x_st | 218 | 6.527 | 7.0317 | 0.04583 |
| hp_hsa-mir-378f_st | 219 | 5.6756 | 5.9562 | 0.04625 |
| hsa-miR-200b-star_st | 221 | 5.2598 | 5.651 | 0.04658 |
| hsa-miR-518b_st | 226 | 7.0275 | 8.1708 | 0.04712 |
| hp_hsa-mir-411_st | 230 | 5.2873 | 5.53 | 0.04793 |
| hsa-miR-4760-3p_st | 233 | 4.2133 | 4.588 | 0.04939 |
| hsa-miR-654-3p_st | 234 | 7.8846 | 8.5192 | 0.04939 |
